# Activation-induced pyroptosis contributes to the loss of MAIT cells in chronic HIV-1 infected patients

**DOI:** 10.1186/s40779-022-00384-1

**Published:** 2022-05-27

**Authors:** Peng Xia, Xu-Dong Xing, Cui-Xian Yang, Xue-Jiao Liao, Fu-Hua Liu, Hui-Huang Huang, Chao Zhang, Jin-Wen Song, Yan-Mei Jiao, Ming Shi, Tian-Jun Jiang, Chun-Bao Zhou, Xi-Cheng Wang, Qing He, Qing-Lei Zeng, Fu-Sheng Wang, Ji-Yuan Zhang

**Affiliations:** 1 Senior Department of Infectious Diseases, the Fifth Medical Center of Chinese PLA General Hospital, National Clinical Research Center for Infectious Diseases, Savaid Medical School, University of Chinese Academy of Sciences, Beijing, 100039 China; 2 Department of Infectious Diseases and Hepatology, the First Affiliated Hospital of Zhengzhou University, Zhengzhou, 450052 China; 3 Biomedical Pioneering Innovation Center (BIOPIC), School of Life Sciences, Peking University, Beijing, 100871 China; 4 Yunnan Infectious Disease Hospital, Kunming, 650301 China; 5 the Third People’s Hospital of Shenzhen, School of Medicine, Southern University of Science and Technology, Shenzhen, 518112 Guangzhou China

**Keywords:** Acquired immune deficiency syndrome, Human immunodeficiency virus, Mucosal-associated invariant T cells, Pyroptosis, Immune reconstitution

## Abstract

**Background:**

Mucosal-associated invariant T (MAIT) cells are systemically depleted in human immunodeficiency virus type 1 (HIV-1) infected patients and are not replenished even after successful combined antiretroviral therapy (cART). This study aimed to identify the mechanism underlying MAIT cell depletion.

**Methods:**

In the present study, we applied flow cytometry, single-cell RNA sequencing and immunohistochemical staining to evaluate the characteristics of pyroptotic MAIT cells in a total of 127 HIV-1 infected individuals, including 69 treatment-naive patients, 28 complete responders, 15 immunological non-responders, and 15 elite controllers, at the Fifth Medical Center of Chinese PLA General Hospital, Beijing, China.

**Results:**

Single-cell transcriptomic profiles revealed that circulating MAIT cells from HIV-1 infected subjects were highly activated, with upregulation of pyroptosis-related genes. Further analysis revealed that increased frequencies of pyroptotic MAIT cells correlated with markers of systemic T-cell activation, microbial translocation, and intestinal damage in cART-naive patients and poor CD4^+^ T-cell recovery in long-term cART patients. Immunohistochemical staining revealed that MAIT cells in the gut mucosa of HIV-1 infected patients exhibited a strong active gasdermin-D (GSDMD, marker of pyroptosis) signal near the cavity side, suggesting that these MAIT cells underwent active pyroptosis in the colorectal mucosa. Increased levels of the proinflammatory cytokines interleukin-12 (IL-12) and IL-18 were observed in HIV-1 infected patients. In addition, activated MAIT cells exhibited an increased pyroptotic phenotype after being triggered by HIV-1 virions, T-cell receptor signals, IL-12 plus IL-18, and combinations of these factors, in vitro.

**Conclusions:**

Activation-induced MAIT cell pyroptosis contributes to the loss of MAIT cells in HIV-1 infected patients, which could potentiate disease progression and poor immune reconstitution.

**Supplementary Information:**

The online version contains supplementary material available at 10.1186/s40779-022-00384-1.

## Background

Human mucosal-associated invariant T (MAIT) cells harbor a semi-invariant T-cell receptor (TCR) that mostly contains the relatively conserved Vα7.2-Jα33 chain, preferentially paired with a restricted Vβ2 or Vβ13 repertoire [1–3], and are characterized by the CD3^+^ CD161^high^ Vα7.2^+^ phenotype [2]. MAIT cells combine microbial metabolic derivatives of the highly conserved riboflavin presented by MR1 (MHC-I-like related molecule), which is critical for the development of MAIT cells [3, 4]. MAIT cells can be effectively activated through TCR signaling in various infective organisms (including *Mycobacteria*, *Pseudomonas*, and *Enterobacter* species) possessing a riboflavin synthesis pathway [5, 6]. In addition to TCR signaling, several cytokine signals, including interleukin-12 (IL-12), IL-15, IL-18, IL-23, and type I interferons, or their combinations [7–11], can induce the activation of MAIT cells. Upon activation, MAIT cells secrete effector molecules, including interferon-γ (IFN-γ), tumor necrosis factor-α (TNF-α), granzyme B, and granulysin to respond to the signaling aroused by viral infection [12, 13]. MAIT cells respond to a variety of viruses in in vitro infection models or in vivo and play protective roles [14] against human viral infections caused by hepatitis B, C, and D viruses, influenza virus, and human immunodeficiency virus type 1 (HIV-1).

Some characteristics of MAIT cells have been investigated in simian immunodeficiency virus (SIV)-infected rhesus macaques and HIV-1 infected humans. Systematic deletion of MAIT cells is the predominant feature of this type of cell during HIV-1/SIV infection. For example, MAIT cells showed a very early expansion in peripheral blood during acute HIV-1 infection [15–17], followed by a profound loss, and the reduced quantity or number of MAIT cells has been reported to be associated with disease progression [16, 17]. In addition, perturbation of MAIT cells in combined antiretroviral therapy (cART)-naive patients is associated with time since HIV-1 diagnosis, immune activation status, and expression of transcription factors T-bet or Eomes in MAIT cells [13, 16]. Importantly, the frequency of MAIT cells fails to restore to normal levels in healthy individuals, even with successful cART. Furthermore, MAIT cells express high levels of activation markers CD69, CD38, and human leukocyte antigen (HLA)-DR in cART-naive patients, as well as CD57, Tim-3, and conjugated with reduced CD27 and CD127 [13, 17, 18], suggesting that they are hyperactivated and functionally exhausted.

Whether the severe loss of MAIT cells is mediated by cell death, tissue trafficking, or loss of cell surface CD161 expression [16, 19] remains controversial. Transcriptome signature analysis revealed that innate natural killer T (iNKT) cells and MAIT cells are deficient in expressing a key transcript factor X-linked inhibitor of apoptosis (XIAP) [20]. Cell biology analysis revealed that MAIT cells exhibit a pro-apoptotic phenotype with elevated expression of promyelocytic leukemia zinc finger, which can decoy the function of XIAP, suggesting that MAIT cells are in a proapoptotic state intrinsically [21]. The costimulatory TNF superfamily receptor OX40 acts as an amplifier for promoting MAIT cell apoptosis [22]. More recently, type I interferons or IFN-α were suggested to promote TCR- or cytokine-mediated MAIT cell activation and subsequent MAIT cell loss [23, 24]. Furthermore, apoptotic MAIT cells have been observed in many in vivo studies, including asthma, dermatomyositis, rheumatoid arthritis, systemic lupus erythematosus [25–27], inflammatory bowel diseases [28], coronary artery disease, type 2 diabetes mellitus [22, 29], and some viral infection [30, 31]. Of note, one study showed that, compared with those from healthy donors, MAIT cells from cART-naive patients have increased activation-induced apoptosis when treated with paraformaldehyde-fixed *Escherichia coli* (*E. coli*) in vitro [17]. In contrast, another study found that MAIT cell loss does not occur as a consequence of apoptosis via active caspase-3 in SIV-infected rhesus macaques [32]. Thus, it is still unclear whether there are other mechanisms beyond the apoptosis paradigm that explain the loss of MAIT cells during chronic HIV-1/SIV infection.

Pyroptosis is a recently defined programmed cell death characterized by active caspase-1-mediated gasdermin-D (GSDMD) cleavage and subsequent plasma membrane instability leading to the emission of proinflammatory signals, including the signature cytokines IL-1β and IL-18 [33, 34]. GSDMD or gasdermin-E (GSDME) in human B leukemic cells can be activated by chimeric antigen receptor T cell-released granzyme B [35] and activated GSDMD is required for an optimal cytotoxic T lymphocyte response to lung cancer cells [36]. Unexpectedly, peripheral CD4^+^ T cells are naturally resistant to pyroptosis [37, 38]. Therefore, the potential clinical significance of MAIT cell loss through pyroptosis during the different stages of HIV-1 disease remains to be elucidated. The aim of this study is to identify the mechanism underlying MAIT cell depletion.

## Methods

### Study subjects and human samples

A total of 127 HIV-1 infected individuals, including 69 treatment-naive patients (TPs), 28 complete responders (CRs), 15 immunological non-responders (INRs), and 15 elite controllers (ECs), were enrolled at the Fifth Medical Center of Chinese PLA General Hospital, Beijing, China. The criteria for diagnosis were defined according to a previous report [39] and exclusion criteria included co-infection with hepatitis B or hepatitis C viruses, tuberculosis, and other opportunistic infections [40]. Briefly, TP individuals (with different peripheral CD4^+^ T cell counts) were newly diagnosed with chronic HIV-1 infection at different stages of disease progression without cART and were ready to receive cART after their 1-week of diagnosis [39, 40]. CRs were defined as individuals who received cART for more than 2 years with peripheral CD4^+^ T cell counts above 250 cells/μl and plasma HIV-1 RNA below the detectable limit, and INRs were defined as individuals who received cART for more than 2 years with peripheral CD4^+^ T cell count below 200 cells/μl and plasma HIV-1 RNA less than the detectable limit [41, 42]. ECs were HIV antibody-positive, but with plasma HIV-1 RNA levels below the detectable limit without receiving cART [40]. The detailed characteristics and cART regimen of the patients are listed in Table 1. For comparison, 33 healthy controls (HCs) were age-matched to the enrolled patients as controls. Colorectal mucosal tissues were obtained from seven biopsies from HIV-1 infected patients and seven biopsies from HIV-1 negative patients who underwent histological diagnosis for severe lower gastrointestinal bleeding and a family history of intestinal polyps. The detailed characteristics of the patients who underwent tissue biopsy are summarized in Table 2. Tissues were fixed in 10% cold neutral buffered formalin and embedded in paraffin. The study design and protocols were approved by the Ethics Committee of the Fifth Medical Center of Chinese PLA General Hospital (2016164D). Written informed consent was obtained from each participant.

**Table 1:** Characteristics of healthy controls and enrolled HIV-1 infected cART-naive or long-term cART-treated patients in flow cytometry phenotyping

Characteristic	HCs (*n* = 33)	ECs (*n* = 15)	TPs		CRs (*n* = 28)	INRs (*n* = 15)
			CD4 ≥ 350 (*n* = 28)	CD4 < 350 (*n* = 41)		
Age [years, *M* (*Q*_1_, *Q*_3_)]	29.0 (27.0, 35.5)	35.0 (29.0, 43.0)	28.5 (22.0, 40.0)	34.0 (27.0, 37.5)	25.0 (22.5, 31.5)	30.0 (24.8, 25.3)
Gender [male, *n*(%)]	18 (54.5)	12 (80.0)	23 (82.1)	33 (80.4)	26 (92.9)	14 (93.3)
CD4^+^ T cell counts [cells/µl, *M* (*Q*_1_, *Q*_3_)]	822.0 (684.0, 981.5)	627.0 (458.0, 741.0)	421.0 (374.3, 596.8)	194.0 (134.0, 277.5)	575.0 (506.5, 725.5)	155.0 (137.5, 178.0)
CD8^+^ T cell counts [cells/µl, *M* (*Q*_1_, *Q*_3_)]	621.0 (512.5, 765.0)	1026.0 (822.0, 1276.0)	1267.0 (1008.0, 1757.0)	1061.0 (676.0, 1335.0)	714.0 (558.5, 826.0)	694.5 (353.5, 893.5)
Plasma viral loads [× 10^4^ copies/ml, *M* (*Q*_1_, *Q*_3_)]	NA	NA	15.8 (3.8, 56.9)	11.3 (4.2, 46.8)	< LOD	< LOD
cART regimen [*n*(%)]						
3TC/EFV/TDF	NA	NA	NA	NA	25 (89.2)	14 (93.3)
3TC/LPV/TDF	NA	NA	NA	NA	2 (7.1)	1 (6.7)
3TC/AZT/TDF	NA	NA	NA	NA	1 (3.6)	0
3TC/DTG/TDF	NA	NA	NA	NA	0	1 (6.7)
cART duration time [month, *M* (*Q*_1_, *Q*_3_)]	NA	NA	NA	NA	42.9 (34.9, 65.8)	46.2 (35.4, 90.3)

*HIV* human immunodeficiency virus, *HCs* healthy controls, *ECs* elite controllers, *TPs* treatment-naive patients, *CRs* complete responders, *INRs* immunological non-responders, *NA* not applicable, *cART* combined antiretroviral therapy, *3TC* lamivudine, *EFV* efavirenz, *TDF* tenofovirdisoproxil, *LPV* lopinavir, *AZT* azidothymidine, *DTG* dolutegravir, < *LOD* below the limit of detection of 80 copies/ml

**Table 2:** Characteristics of HIV-1 negative controls and HIV-1 infected patients with colorectal tissue biopsies

HIV status/patient ID	Gender	Age (years)	Comorbidities	CD4^+^ T cell count (cells/μl)	Plasma viral load (copies/ml)	cART regimen
HIV-1 negative controls (*n* = 7)						
0057	M	49	Colon polyp	NA	NA	NA
7403	M	48	Rectum polyp	NA	NA	NA
7435	M	55	Colon polyp	NA	NA	NA
7559	F	70	Colon polyp	NA	NA	NA
7582	F	50	Colon polyp	NA	NA	NA
7590	M	46	Colon polyp	NA	NA	NA
7894	F	54	Colon polyp	NA	NA	NA
HIV-1 infected patients (*n* = 7)						
0118	F	40	Colon cancer	435	< LOD	3TC/AZT/EFV
0128	M	55	Rectum cancer	86	693,800	Untreated
0129	F	61	Colon cancer	246	< LOD	3TC/AZT/EFV
0288	M	36	Colon cancer	119	< LOD	3TC/AZT/NVP
0293	M	63	Colon cancer	327	172,500	Untreated
0295	M	31	Colon cancer	913	< LOD	3TC/EFV/TDF
0316	F	53	Colon cancer	338	< LOD	3TC/EFV/TDF

*HIV* human immunodeficiency virus, *cART* combined antiretroviral therapy, *NA* not applicable, < *LOD* below the limit of detection of 80 copies/ml, *3TC* lamivudine, *AZT* azidothymidine, *EFV* efavirenz, *NVP* nevirapine, *TDF* tenofovirdisoproxil

### Immunophenotyping of MAIT cells

MAIT cells were identified as CD161^high^-TCR Vα7.2^+^ cells in CD3^+^ T cells and gating strategies are shown in Additional file 1: Fig. S1. Detailed information of the antibodies is provided in Additional file 2: Table S1. Peripheral blood mononuclear cells (PBMCs) were harvested from fresh peripheral blood of all enrolled subjects, labeled with the above-mentioned antibodies on ice for 30 min, and then thoroughly washed and fixed for further analysis.

### Activation of MAIT cells

Fresh PBMCs were resuspended at a density of 1 × 10^6^ cells/ml in conditioned medium with 2 mmol/L L-glutamine, 10% fetal bovine serum, and 100 U/ml penicillin and streptomycin. PBMCs were stimulated at 37 ℃ and 5% CO_2_ with: (1) Medium only or HIV-1 particles (HIV-1 R5 strain JR-CSF stocks, HIV RNA titer: 6 × 10^9^ copies/μl) for 48 h. (2) Medium only or αCD3/CD28 mAb (both are 1 µg/ml, both are from Thermo Fisher Scientific, USA) or αCD3/CD28 mAb (both are 1 µg/ml) plus HIV-1 particles for 24 h. (3) Medium only or recombinant human IL-12 p70 (50 ng/ml, PeproTech, USA) plus IL-18 (50 ng/ml, R&D Systems, USA) for 24 h in the absence or presence of HIV-1 particles. (4) Medium only or 1% PFA-fixed *E. coli* DH5α (a gift from Professor Lu Wang’s lab) at a multiplicity of infection of 10 for 24 h in the absence or presence of HIV-1 particles.

For intracellular cytokine detection, GolgiStop (1 µg/ml, BD Biosciences, USA) was added during the stimulation process. Cells were harvested to stain surface markers, followed by cell fixation and permeabilization (BD Bioscience, USA) for 30 min, incubated with intracellular anti-IFN-γ and TNF-α antibodies on ice for 1 h, and then washed for flow cytometry analysis.

For fluorescently labeled inhibitor of caspases (FLICA) caspase-1 detection, FLICA staining was performed according to the manufacturer’s instructions (Bio-Rad, USA). Cells were incubated with FLICA caspase-1 reagent for 1 h at 37 ℃ and cells were washed for downstream surface marker staining and flow cytometry analysis. Flow cytometry acquisition was performed on a BD FACSVerse flow cytometer driven by FACSDiva software (BD Biosciences, USA). At least 5 × 10^5^ cells were acquired per run. Data analyses were performed using FlowJo version 10.5 software (TreeStar, USA).

### Single-cell RNA sequencing (scRNA-seq) data processing, multiple dataset integration, and cell-type annotation

Gene expression matrices of each sample were generated using Cell Ranger (version 3.0.2) Pipeline coupled with the human reference version GRCh38. The output filtered gene expression matrices were analyzed using R software (version 3.5.3) with the Seurat package (version 3.0.0) [41]. In brief, we initialized the Seurat object with the CreateSeuratObject function with genes expressed > 0.1% of the data and cells with > 200 genes detected. We selected and filtered the cells based on quality control (QC) metrics. Low-quality cells were removed if they met the following criteria: (1) < 1000 unique molecular identifiers, (2) < 500 genes, or (3) > 10% unique molecular identifiers derived from the mitochondrial genome. After removal of low-quality cells, the gene expression matrices were normalized using the NormalizeData function, and 2000 features with high cell-to-cell variation were calculated using the FindVariableFeatures function. To reduce the dimensionality of the datasets, the RunPCA function was conducted with default parameters on linear transformation scaled data generated by the ScaleData function. ElbowPlot, DimHeatmap, and JackStrawPlot functions were used to identify the true dimensionality of each dataset, as recommended by Seurat developers. Finally, we clustered cells using FindNeighbors and FindClusters functions and performed non-linear dimensional reduction with the RunUMAP function with default settings. All details of the Seurat analyses performed in this work can be found in the website tutorial (https://satijalab.org/seurat/v3.0/pbmc3k_tutorial.html). To compare cell types and proportions in T cells from HIV-infected patients and HCs across different datasets, we employed the integration methods described previously (https://satijalab.org/seurat/v3.0/integration.html) [42]. The Seurat package (version 3.0.0) was used to assemble multiple distinct scRNA-seq datasets into an integrated and unbatched dataset. In brief, we identified 2000 features with high cell-to-cell variation, as described above. Second, we identified “anchors” between individual datasets with the FindIntegrationAnchors function and inputted these “anchors” into the IntegrateData function to create a “batch-corrected” expression matrix of all cells, which allowed cells from different datasets to be integrated and analyzed together.

### Cell type annotation, differential expression genes (DEG) identification, and functional enrichment

After non-linear dimensional reduction and projection of all cells into a two-dimensional space by UMAP, cells were clustered together according to common features. The FindAllMarkers function in Seurat was used to identify markers for each of the identified clusters. Clusters were then classified and annotated based on the expression of canonical markers of particular cell types. Clusters expressing two or more canonical cell-type markers were classified as doublet cells and excluded from further analyses. In this way, we identified MAIT cells and extracted the MAIT cluster for further analysis. To identify DEGs across different sample conditions, the FindAllMarkers function in Seurat was used and genes with *P*-value < 0.01 were identified. Functional enrichment analyses and gene set enrichment analysis (GSEA) for identified gene sets were performed using ClusterProfiler (R package, version 3.10.1) [43]. Gene sets were derived from the GO Biological Process Ontology.

### Data and code availability

We have deposited our data in the GSA database http://bigd.big.ac.cn/gsa-human under HRA000190 and a detailed description of our dataset is available at https://bigd.big.ac.cn/gsa-human/s/33pJKjg6. Custom scripts for analyzing the data are available upon request.

### Human tissue processing, histopathology, and immunohistochemistry

Tissue sections (4 µm) were stained with primary antibodies: anti-MDR1, anti-IL-18Rα, anti-TCR Vα7.2, anti-CD4, anti-active GSDMD (a gift from Professor Feng Shao’s lab) [40], and detailed information on the antibodies is provided in Additional file 2: Table S2. The avidin–biotin system with alkaline phosphatase (blue color) and the non-biotin system with enhanced 3,3-N-diaminobenzidine tetrahydrochloride (brown color) as substrates were used to perform double staining. MDR1^+^ cells or IL-18Rα^+^TCR Vα7.2^+^ cells in the colon mucosa were identified as MAIT cells. The absolute number of MAIT cells, pyroptotic MAIT cells (MDR1^+^GSDMD^+^ double-positive cells), and CD4^+^ MAIT cells (CD4^+^MDR1^+^ double-positive cells) in tissues were independently counted by two pathologists from three representative high-power fields (hpf, ×400) in the corresponding anatomical position of the colorectal mucosa from HIV-1 negative controls and HIV-1 infected patients.

### HIV-1 RNA quantification and CD4 T cell count

Plasma HIV-1 RNA was qualified and quantified by HIV-1 real-time PCR assays V2 (Qiagen, Germany) and CFX96 real-time system (Bio-Rad, USA). The cut-off value of the assays was 80 copies/ml. Peripheral blood CD4^+^ T cell counts were counted using FACSCount reagent kits (BD Biosciences, USA) based on flow cytometry.

### ELISA

The soluble CD14 (sCD14), intestinal fatty acid-binding protein (I-FABP), IL-12p70, and IL-18 kits (all purchased from R&D Systems, USA) were used to quantify the plasma concentrations of sCD14, I-FABP, IL-12p70, and IL-18 according to the manufacturer’s protocols. All samples were analyzed in duplicates.

### Statistical analysis

Data were represented as *M*(*Q*_1_, *Q*_3_) and were analyzed using GraphPad Prism software (version 8.0; GraphPad Software). Correlations between variables were evaluated using the Spearman rank correlation test. Mann–Whitney *U* tests were used to compare the two groups. The Wilcoxon signed-rank test was used for matched pairs. All tests were two-tailed and *P*-value < 0.05 was considered.

## Results

### Loss of MAIT cells is correlated with markers of disease progression, systemic T cell activation, microbial translocation, and gut mucosa damage in cART-naive patients

**Fig. 1:**
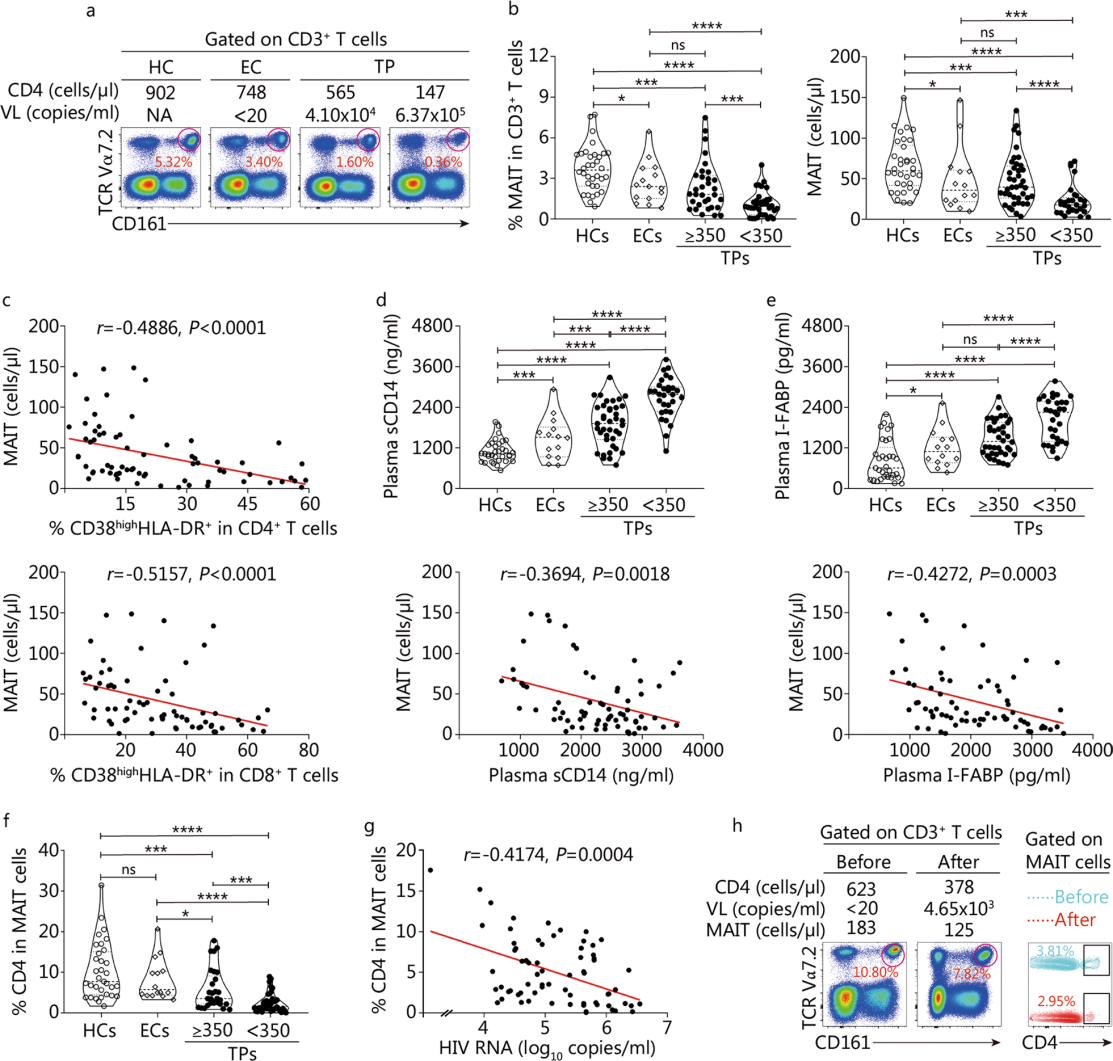
A decrease in mucosal-associated invariant T (MAIT) cells correlates with markers for disease progression, systemic T cell activation, microbial translocation, and gut mucosa damage in combined antiretroviral therapy (cART)-naive patients. **a** Representative FACS plots from one healthy control (HC), one elite controller (EC), one treatment-naive patient (TP) with CD4^+^ T cell count ≥ 350 cells/µl and another TP with CD4^+^ T cell count < 350 cells/µl showing MAIT cells (defined as CD161^high^TCR Vα7.2^+^, gated on live, doublet-excluded CD3^+^ T cells). **b** Pooled data showing MAIT-cell percentages and absolute numbers from HCs (hollow circles, *n* = 33), ECs (diamonds, *n* = 14), TPs (solid circles, total *n* = 69: CD4^+^ T cell count ≥ 350 cells/µl subgroup, *n* = 34; CD4^+^ T cell count < 350 cells/µl subgroup, *n* = 35). **c** Correlation between MAIT-cell absolute numbers and percentages of CD38^high^HLA-DR^+^-expressing CD4^+^ T cells (top panel) and CD38^high^HLA-DR^+^-expressing CD8^+^ T cells (bottom panel) in TPs (*n* = 69). Plasma sCD14 levels (**d**, top panel) and I-FABP levels (**e**, top panel) in HCs (*n* = 33), ECs (*n* = 14) and TPs (*n* = 69). Correlation between MAIT-cell absolute numbers and plasma sCD14 levels (**d**, bottom panel) or I-FABP levels (**e**, bottom panel) in TPs (*n* = 69). **f** Pooled data showing the proportion of CD4^+^ MAIT cells in total MAIT cells in HIV-1 infected patients. **g** Correlation between plasma HIV-1 viral load and proportion of CD4^+^ MAIT cells in TPs (*n* = 69). **h** FACS plots showing MAIT cells and corresponding CD4^+^ subset from one EC before and after spontaneous loss of control of HIV-1 replication. Each symbol represents a single individual. Data are expressed as *M* (*Q*_1_, *Q*_3_). Mann–Whitney *U* test (**b**, **d**, **e** and **f**). Spearman’s correlation test (**c**, **d**, **e** and **g**). *P*-value and Spearman’s Rho value are shown. *P < 0.05, ^***^P < 0.001, ^****^P < 0.0001, ns non-signifcant. VL viral load, NA not available, TCR T-cell receptor, HLA human leukocyte antigen, sCD14 soluble CD14, I-FABP intestinal fatty acid-binding protein

We detected the profile of MAIT cells in peripheral blood CD3^+^ T cells from a cohort of HIV-1 infected patients and found that both the frequencies and absolute numbers of MAIT cells were dramatically decreased in TPs compared to HCs (*P* < 0.01, Fig. 1a, b). Further analysis indicated that a more profound loss of MAIT cells was observed in TPs with CD4^+^ T cell count < 350 cells/µl than that in TPs with CD4^+^ T cell count ≥ 350 cells/µl (*P* < 0.001, Fig. 1b). In combination with a previous report [16], our data confirmed that the loss of MAIT cells was correlated with disease progression. Although we did not observe obvious correlations between the frequencies of MAIT cells with CD4^+^ T cell counts (Additional file 1: Fig. S2a) and HIV viral load (Additional file 1: Fig. S2b) in TPs, we found that the frequencies of MAIT cells were negatively correlated with the frequencies of CD38^high^HLA-DR^+^-expressing MAIT cells (*r* =  − 0.3364, *P* = 0.0003, Additional file 1: Fig. S2c). We further found that both the frequencies and absolute numbers of MAIT cells in ECs were decreased when compared with HCs (*P* < 0.05, Fig. 1b), suggesting that the loss of circulating MAIT cells occurred even in ECs.

When TPs were considered in the analysis, it was shown that the absolute numbers of TP-MAIT cells were negatively correlated with the frequencies of CD38^high^HLA-DR^+^-expressing CD4^+^ T cells (*r* =  − 0.4886, *P* < 0.0001, Fig. 1c**)** and the frequencies of CD38^high^HLA-DR^+^-expressing CD8^+^ T cells (both markers of systemic T cell activation) (*r* =  − 0.5157, *P* < 0.0001, Fig. 1c).

We next measured the levels of sCD14, a marker of monocyte/macrophage activation, in the plasma of HCs, ECs, and TPs. The plasma level of sCD14 was significantly elevated in TPs, especially in patients with CD4^+^ T cell count < 350 cells/µl (*P* < 0.0001, Fig. 1d). Correlation analysis revealed that the absolute numbers of MAIT cells were negatively correlated with plasma sCD14 levels in TPs (*r* =  − 0.3694, *P* = 0.0018, Fig. 1d).

We next determined the levels of I-FABP, a marker of intestinal mucosa damage. The I-FABP levels were increased in the TPs compared with those in the HCs, and a significant stepwise increase in the levels of I-FABP was observed in patients with CD4^+^ T cell count < 350 cells/µl (*P* < 0.0001, Fig. 1e). Further analysis showed that the absolute numbers of MAIT cells were negatively correlated with the levels of plasma I-FABP in total TPs (*r* =  − 0.4272, *P* = 0.0003, Fig. 1e). Given that MAIT cells play a pivotal role in defending microbe invasion and tissue repair in mucosal tissues [44], these data suggest that reduced numbers of MAIT cells may decrease the ability to defend microbial translocation and repair gut mucosa in TP individuals.

CD4^+^ MAIT cells are a subset of MAIT cells that probably serve as a target of HIV-1 [45]. We found that the percentages of CD4^+^ MAIT cells significantly decreased in MAITs from ECs and TPs (*P* < 0.05 or *P* < 0.0001, Fig. 1f). Interestingly, the percentages of CD4^+^ MAIT cell compartments (not CD4^–^ MAIT cell compartments) in total MAIT cells was significantly correlated with plasma HIV viral load in TPs (*r* =  − 0.4174, *P* = 0.0004, Fig. 1g). We found that the frequency and an absolute number of MAIT cells, as well as CD4^+^ MAIT cells, were decreased in one EC patient after losing control of HIV-1 replication (Fig. 1h).

Taken together, these data enrich the clinical characteristics of the profound depletion of peripheral blood MAIT cells in patients with chronic HIV-1 infection.

### scRNA-seq revealed that MAIT cells exhibit a distinct transcriptional profile associated with activation and pyroptosis

**Fig. 2:**
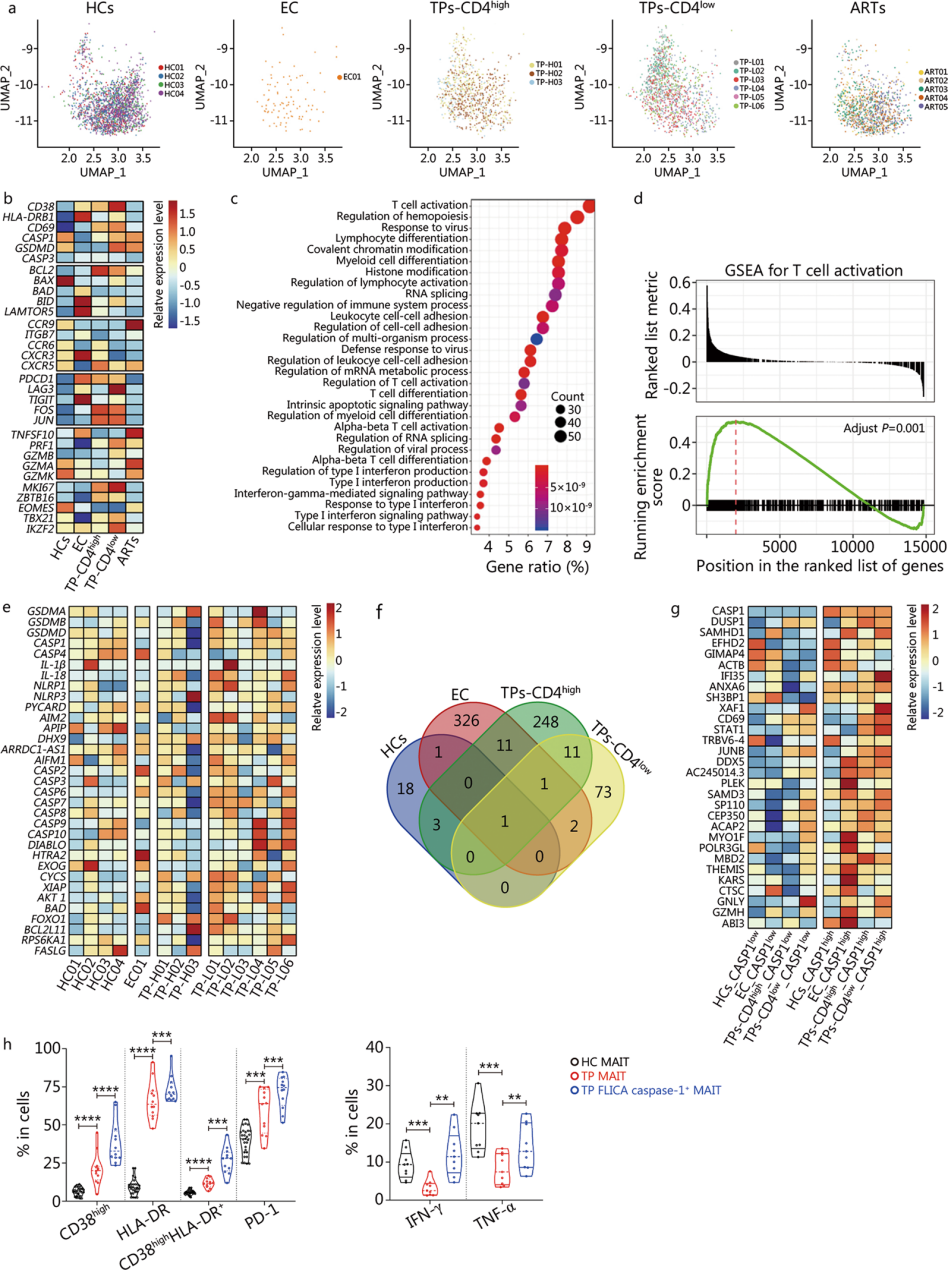
scRNA-seq analyses reveal the transcriptional characterization of mucosal-associated invariant T (MAIT) cells in human immunodeficiency virus (HIV)-infected patients. **a** UMAP projection of 5632 MAIT cells from 5 different sample conditions. Each dot corresponds to a single cell, colored according to different samples. **b** Heatmap showing selected markers’ relative expression levels in different conditions. Columns denote sample conditions, and rows denote genes. **c** A dot plot showing the Gene Ontology enrichment results for differentially expressed genes identified by comparing TPs with HCs. The top 30 GO terms with *P*-value cut-off = 0.01 and *Q*-value cut-off = 0.05 were shown. **d** GSEA enrichment results for direct T cell activation. *P*-values were calculated by GSEA with the Benjamin-Hochberg correction. **e** Heatmap showing selected markers’ relative expression levels in different samples. Columns denote samples, and rows denote genes. **f** A Venn diagram showing the genes shared by four conditions. **g** Heatmap showing the expression of shared genes of (**f**) under at least two conditions. **h** Pooled data showing expression of indicated markers in HC-MAIT, TP-MAIT (*n* = 9) and TP FLICA caspase-1^+^ MAIT (*n* = 9) cells. Each symbol represents a single individual. Data are expressed as *M* (*Q*_1_, *Q*_3_). Mann–Whitney *U* test (**h**). ^**^P < 0.01, ^***^P < 0.001, ^****^P < 0.0001. HCs healthy controls, EC elite controller, TPs treatment-naive patients, ART antiretroviral therapy, GSEA gene set enrichment analysis, PD-1 programmed death-1, IFN-γ interferon-γ, TNF-α tumor necrosis factor-α

We performed droplet-based scRNA-seq (10× genomics) to study the transcriptomic profiles of MAIT cells in HIV-infected patients. We included five conditions: HCs (*n* = 4), EC (*n* = 1), TPs-CD4^high^ (*n* = 3), TPs-CD4^low^ (*n* = 6), and ARTs (*n* = 5) (Fig. 2a). The characteristics of the enrolled HIV-1 infected patients are summarized in Additional file 2: Table S3. Magnetic cell sorting-purified CD4^+^ and CD8^+^ T cells were used to perform scRNA-seq (Additional file 1: Fig. S3a). After QC, we defined one cluster as MAIT cells for unique and broad expression of markers SLC4A10 and TRAV1-2 (Additional file 1: Fig. S3b, c) [46, 47] and obtained 5632 high-quality MAIT cells assigned to five conditions for further analyses (Fig. 2a, Additional file 1: Fig. S3c).

We first investigated the expression of some canonical markers across the sample conditions. Genes related to T cell activation (*CD38*, *HLA-DRB1*, and *CD69*), pyroptosis (*CASP1* and *GSDMD*), and T cell proliferation markers (*MKI67*) were higher in HIV-1 infected patients than in HCs (Fig. 2b). Homing-related markers, such as C–C motif chemokine receptor (CCR)9, CCR6, and CXC-chemokine receptor (CXCR)3, had the lowest expression levels in the TPs-CD4^low^ group among the five conditions (Fig. 2b). It is worth noting that the expression levels of T cell exhaustion markers (PDCD1, LAG3, and TIGIT) in TPs were higher than those in HCs and were downregulated in cART-treated patients, whereas the expression levels of T cell cytotoxicity markers (TNFSF10, PRF1, GZMB, GZMA, and GZMK) in TPs were lower than in HCs and were upregulated in cART-treated patients (Fig. 2b).

A direct comparison between TPs and HCs identified 677 DEGs. Gene ontology functional enrichment showed that MAIT functions related to T cell activation, defense response to the virus, and regulation of lymphocyte activation (Fig. 2c) were significantly upregulated in HIV-1 infected individuals. For cross-validation, GSEA was performed on T cell activation directly (Fig. 2d). We observed significant gene enrichment of T cell activation in HIV-1 infected patients (adjusted *P* = 0.001), and higher levels of pyroptosis-related genes were found in HIV-1 infected patients compared with HCs (Fig. 2e), including genes promoting inflammasome assembly (*NLRP1*, *NLRP3*, and *AIM2*) and genes executing pyroptosis (*GSDMA*, *GSDMB*, *GSDMD*, *CASP1*, and *IL-1β*).

MAIT cells in HCs, ECs, TPs-CD4^low^, and TPs-CD4^high^ conditions were divided into two groups based on *CASP1* expression level (CASP1^high^: ≥ 0.5; CASP1^low^: ≤ 0.1) (see Methods). DEGs were identified by comparing CASP1^high^ to CASP1^low^ MAIT cells under each condition (Fig. 2f). Genes shared under at least two conditions were collected and are shown in Fig. 2g. We found that genes related to T cell activation, HIV-1 restriction, and cytotoxicity, such as *CD69, DUSP1, SAMHD1, IFI35, STAT1, THEMIS, JUNB, CTSC, GNLY*, and *GZMH*, were highly expressed in CASP1^high^ group MAIT cells in each condition, suggesting that higher *CASP1* expression may correlate with higher activation status and higher effector functions in MAIT cells.

Next, we used flow cytometry to validate the expression of the activation markers. In agreement with previous studies, we observed that TP-MAIT cells showed higher levels of CD38 and HLA-DR expression than HC-MAIT cells (*P* < 0.0001, Fig. 2h). Interestingly, FLICA caspase-1^+^ MAIT cells represented ongoing pyroptotic MAIT cells [33, 48, 49]. We found that FLICA caspase-1^+^ TP-MAIT cells displayed a greater increase in CD38 and HLA-DR expression than total TP-MAITs. In addition, FLICA caspase-1^+^ TP-MAIT cells expressed higher levels of programmed death-1 (PD-1) than total TP-MAIT cells (*P* < 0.001, Fig. 2h). Furthermore, decreased IFN-γ and TNF-α expression was observed in TP-MAIT cells in response to αCD3 + αCD28 stimulation when compared with HC-MAIT cells, whereas FLICA caspase-1^+^ TP-MAIT cells produced higher levels of IFN-γ and TNF-α than total TP-MAIT cells (*P* < 0.001, Fig. 2h). These flow cytometry data verify that TP-MAIT cells harbor activation phenotypes and are functionally impaired and strengthen the finding that active MAIT cells from TPs are in a state of hyper-activation. Altogether, MAIT cells exhibited a distinct transcriptional profile linking activation status with pyroptosis both in HCs and in chronic HIV-1 infected patients.

### Increased pyroptotic MAIT cells are correlated with loss of MAIT cells and markers of disease progression, systemic T cell activation, microbial translocation, and mucosal damage in cART-naive patients

**Fig. 3:**
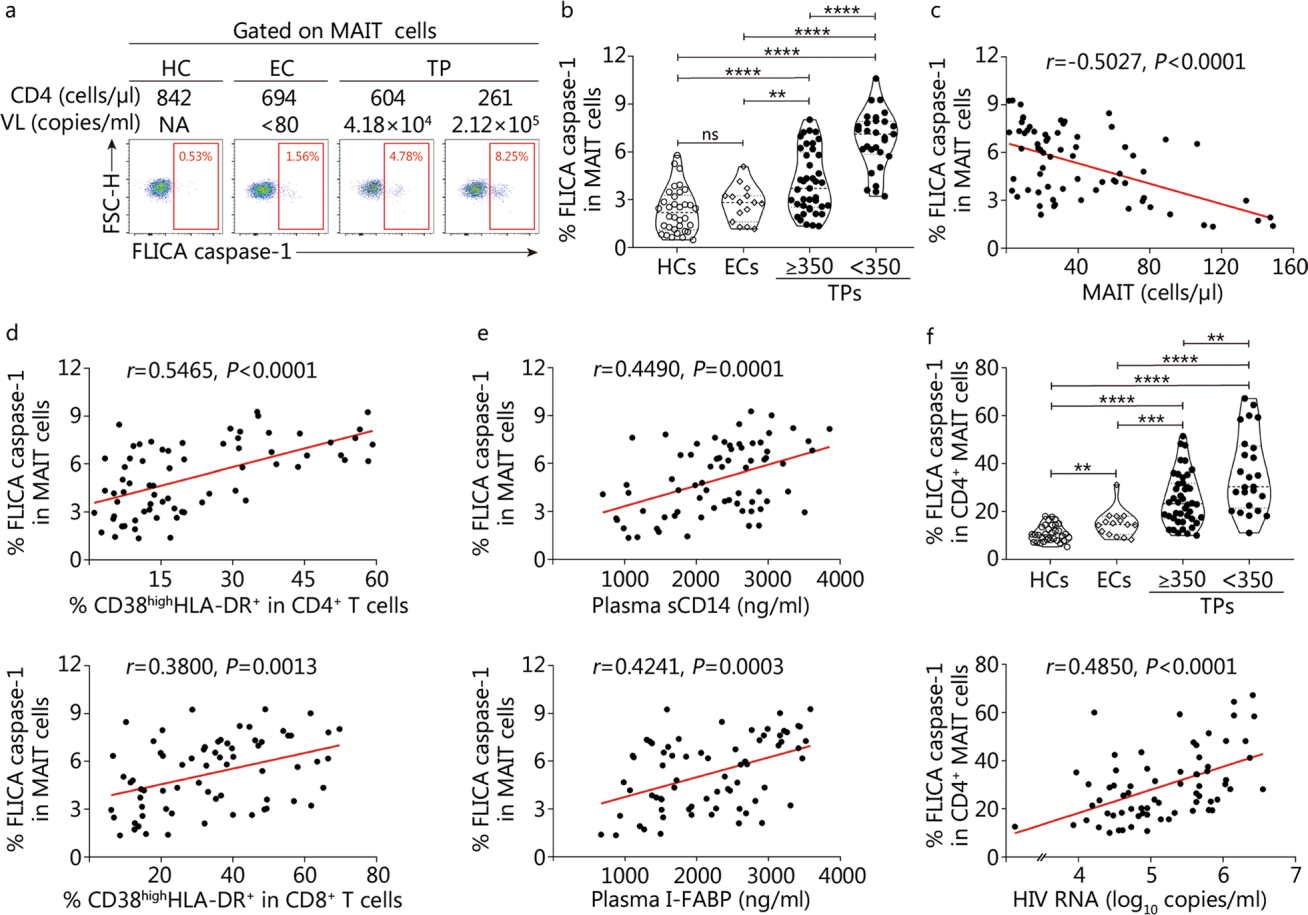
Increased pyroptosis among mucosal-associated invariant T (MAIT) cells is associated with MAIT-cell loss and disease progression in combined antiretroviral therapy (cART)-naive patients. **a** Representative FACS plots from one HC, one EC, one TP with a CD4^+^ T cell count ≥ 350 cells/µl, and another TP with CD4^+^ T cell count < 350 cells/µl showing FLICA-caspase-1^+^ MAIT-cells. **b** Pooled data showing the frequencies of FLICA-caspase-1^+^ MAIT cells from HCs (hollow circles, *n* = 33), ECs (diamonds, *n* = 15), TPs (solid circles, total *n* = 69, CD4^+^ T cell count ≥ 350 cells/µl subgroup *n* = 34, CD4^+^ T cell count < 350 cells/µl subgroup *n* = 35). **c** The relationship between FLICA-caspase-1^+^ MAIT cell frequencies and MAIT cell counts in TPs (*n* = 69). **d** Correlations between FLICA-caspase-1^+^ MAIT cell frequencies and frequencies of CD38^high^HLA-DR^+^–expressing CD4^+^ T cells (top panel) and CD38^high^HLA-DR^+^-expressing CD8^+^ T cells (bottom panel) in TPs (*n* = 69). **e** Correlation analysis between FLICA-caspase-1^+^ MAIT cells and plasma sCD14 levels (top panel) or I-FABP levels (bottom panel) in TPs (*n* = 69). **f** Pooled data showing frequencies of FLICA-caspase-1^+^ cells in CD4^+^ MAIT cells in the above four groups (top panel). Correlation between plasma HIV-1 viral load and frequencies of caspase-1 expression in CD4^+^ MAIT cells in TPs (*n* = 69, bottom panel). Each symbol represents a single individual. Data are expressed as *M* (*Q*_1_, *Q*_3_). Associations were evaluated by using Spearman rank correlation test. Mann–Whitney *U* test (**b** and **f**). Spearman’s correlation test (**c**, **d**, **e** and **f**). *P*-value and Spearman’s Rho value are shown. ^**^P < 0.01, ^***^P < 0.001, ^****^P < 0.0001, ns non-signifcant. HC healthy control, EC elite controller, TP treatment-naive patient, NA not available, FSC-H forward scatter-height, FLICA fluorescently labeled inhibitor of caspases, HLA human leukocyte antigen, sCD14 soluble CD14, I-FABP intestinal fatty acid-binding protein, HIV human immunodeficiency virus

Next, we detected FLICA caspase-1 expression in MAIT cells from HCs and HIV-1 infected patients (Fig. 3a). We found that the frequencies of FLICA caspase-1^+^ MAIT cells in peripheral blood increased greatly in TP individuals, particularly for MAITs of TPs with CD4^+^ T cell count < 350 cells/µl, which expressed a higher level of FLICA caspase-1 than that of TPs with CD4^+^ T cell count ≥ 350 cells/µl (*P* < 0.0001, Fig. 3b). Furthermore, we found that the frequencies of FLICA caspase-1^+^ MAIT cells in TPs were negatively correlated with the absolute numbers of circulating MAIT cells (*r* =  − 0.5027, *P* < 0.0001, Fig. 3c). Further analysis showed that the frequencies of FLICA caspase-1^+^ MAIT cells in TPs were positively correlated with the frequencies of CD38^high^HLA-DR^+^-expressing CD4^+^ T cells, CD38^high^HLA-DR^+^-expressing CD8^+^ T cells, plasma sCD14 levels, and plasma I-FABP levels (*r* = 0.5465, *P* < 0.0001; *r* = 0.3800, *P* = 0.0013;* r* = 0.4490, *P* = 0.0001; *r* = 0.4241, *P* = 0.0003; Fig. 3d, e).

We observed that the frequencies of FLICA caspase-1^+^ cells in CD4^+^ MAIT cells were significantly increased in ECs and TPs compared to HC individuals (*P* < 0.01 or *P* < 0.0001, Fig. 3f). Particularly in TP patients with CD4^+^ T cell count < 350 cells/µl, CD4^+^ MAIT cells exhibited the highest levels of FLICA caspase-1 expression. Importantly, the frequencies of FLICA caspase-1^+^ cells in CD4^+^ TP-MAIT cells were positively correlated with the plasma HIV-1 viral load in TPs (*r* = 0.4850, *P* < 0.0001, Fig. 3f).

Overall, these data confirm the assumption that MAIT cell pyroptosis is associated with circulating MAIT-cell loss and disease progression during chronic HIV-1 infection.

### MAIT cells accumulate in colorectal tissue of HIV-1 infected patients

Intestinal chemotaxis is one of the functional characteristics of MAIT cells. In this study, we examined the expression of gut-homing receptors, integrin α4β7 (abbreviated as B7), and CCR9 [50], in circulating MAIT cells in TP patients and HCs (Additional file 1: Fig. S4a). CCR9^–^B7^high^ MAIT cells increased significantly in TPs but CCR9^+^B7^high^ (small intestine-homing) MAIT cells decreased (*P* < 0.0001, Additional file 1: Fig. S4b).

To examine the absolute numbers and tissue distribution of MAIT cells in the gut mucosa of HIV-1 infected patients and HIV-1 negative controls, we stained MDR1^+^ (substitute marker for MAIT) cells [17] in colorectal tissues from HIV-1 infected patients and HIV-1 negative controls. As shown in Additional file 1: Fig. S4c, MDR1^+^ cells were obvious in colorectal mucosa from HIV-1 negative controls, whereas more abundant MDR1^+^ cells were observed in colorectal mucosa from HIV-1 infected patients. Quantitative analysis of the absolute numbers of MDR1^+^ cells per high-power field showed that colorectal mucosa from HIV-1 infected patients exhibited more MDR1^+^ cells than the colorectal mucosa from HIV-1 negative controls (*P* < 0.05, Additional file 1: Fig. S4c).

To validate this finding, we further examined IL-18Rα^+^TCR Vα7.2^+^ double-positive cells in colorectal tissues, which are usually regarded as tissue-resident MAIT cells [6]. We also observed that the number of IL-18Rα^+^TCR Vα7.2^+^ double-positive cells per high-power field was increased in HIV-1 infected patients vs. HIV-1 negative controls (*P* < 0.0001, Additional file 1: Fig. S4d). These data indicate that MAIT cells were significantly increased in the colorectal mucosa of chronic HIV-1 infected patients.

### MAIT cells undergo active pyroptosis in colorectal mucosa of chronic HIV-1 infected patients

**Fig. 4:**
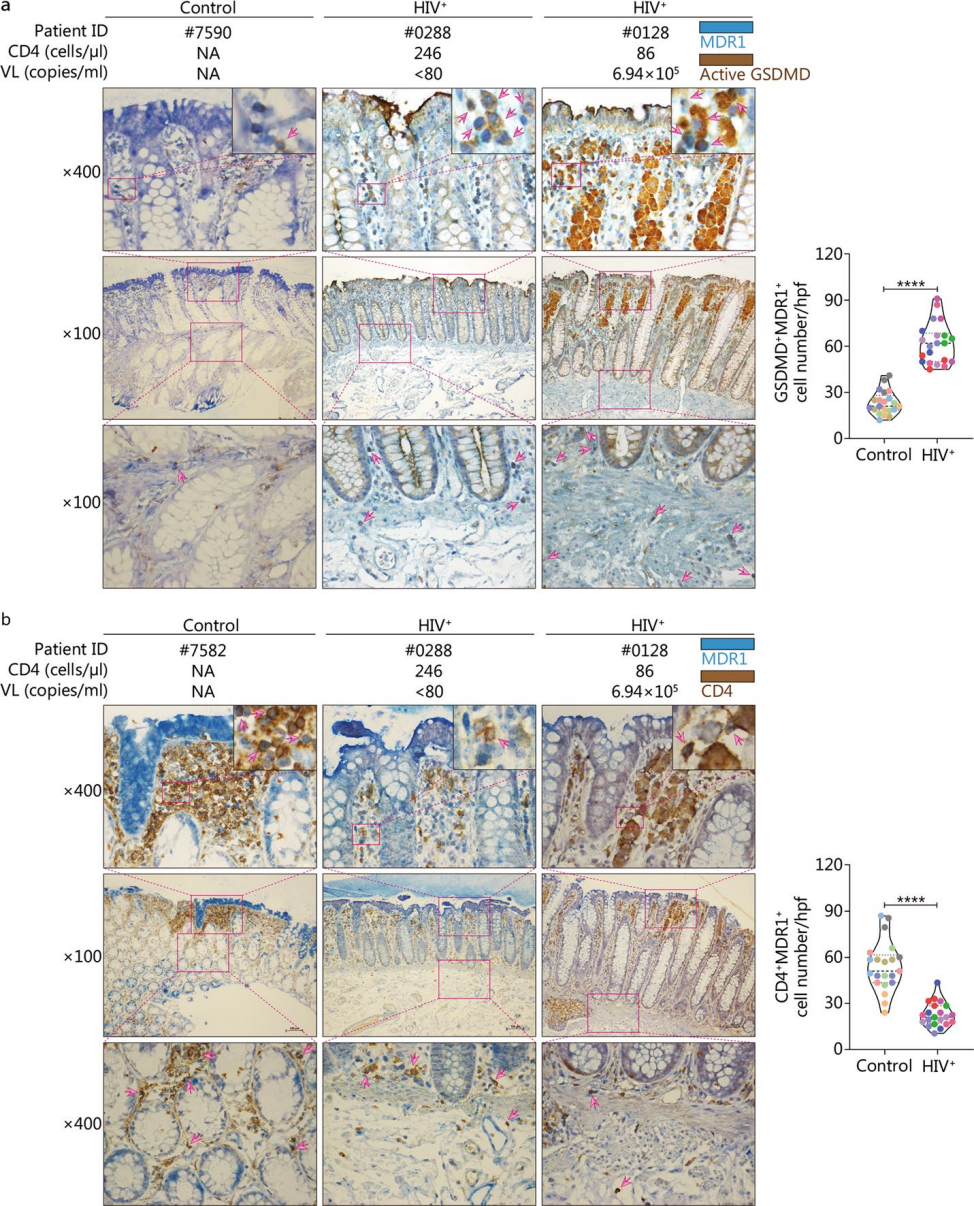
Colorectal mucosa mucosal-associated invariant T (MAIT) cells upregulate active GSDMD expression in human immunodeficiency virus type 1 (HIV-1) infected patients. **a** Representative immunohistochemistry images and quantification of pyroptotic MAIT cells in colorectal tissue from HIV-negative controls (*n* = 7) and HIV^+^ patients (*n* = 7) co-stained with anti-MDR1 and anti-active GSDMD. **b** Representative immunohistochemistry images and quantification of CD4^+^ MAIT cells in colorectal mucosa tissues from HIV-negative controls (*n* = 7) and HIV^+^ patients (*n* = 7) co-stained with anti-MDR1 and anti-CD4. Each color in the pooled data of immune single or double stains represents one single subject, and each dot represents positive cell numbers counted in a single high-power field (hpf, × 400). Arrowheads highlight canonical active GSDMD^+^MDR1^+^ double-positive or CD4^+^MDR1^+^ double-positive cells. Data are expressed as *M* (*Q*_1_, *Q*_3_). ^****^P < 0.0001. Mann–Whitney *U* test (**a**, **b**). VL viral load, NA not available, GSDMD gasdermin-D

Using double immunostaining, we found that GSDMD^+^MDR1^+^ double-positive cells (with diffuse cell swelling) in the gut mucosa of HIV-1 infected patients were mainly localized in the intestinal cavity side of intestinal villi, while GSDMD^+^MDR1^+^ double-positive cells from HIV-1 negative controls were randomly distributed in the intestinal villi (Fig. 4a). Interestingly, more GSDMD^+^MDR1^+^ double-positive cells were detected in patients with detectable plasma viral load than in patients without detectable viral load (Fig. 4a, right panel). In comparison with HIV-1 negative controls, the number of GSDMD^+^MDR1^+^ cells per high-power field was significantly increased in HIV-1 infected patients (*P* < 0.0001, Fig. 4a, pooled data). We detected the numbers of CD4^+^MDR1^+^ double-positive cells in the colorectal mucosa of HIV-1 negative controls and HIV-1 infected patients. Quantitative analysis showed a dramatic decrease in CD4^+^MDR1^+^ double-positive cells in HIV-1 infected patients when compared with HIV-1 negative controls (*P* < 0.0001, Fig. 4b, pooled data).

These data suggest that colorectal mucosa MAIT cells from chronic HIV-1 infected patients undergo active pyroptosis in comparison with those of HIV-1 negative controls in the gut mucosa.

### Activation-induced pyroptosis of MAIT cells may contribute to the loss of MAIT cells in cART-naive patients

**Fig. 5:**
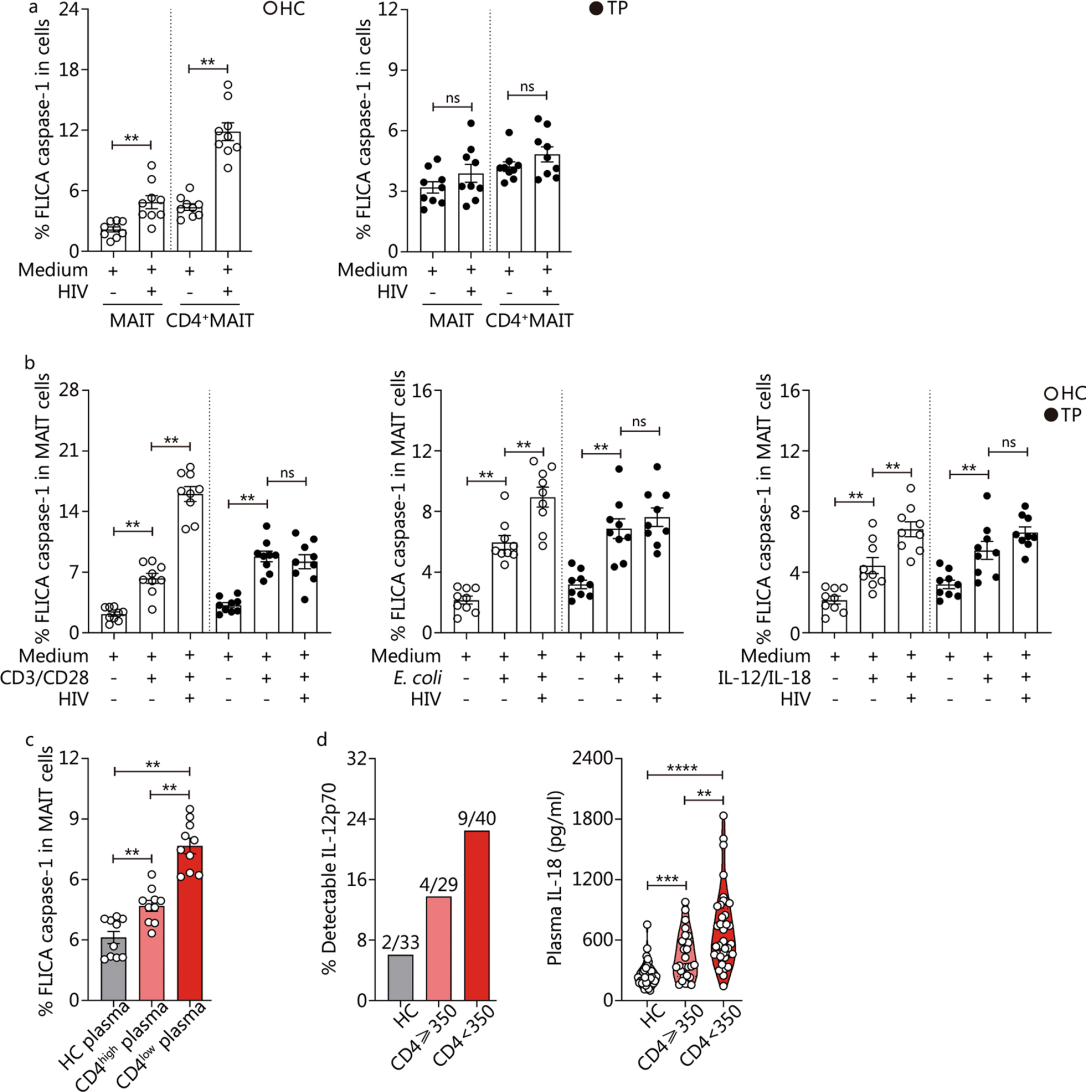
Activation-induced pyroptosis of mucosal-associated invariant T (MAIT) cells is associated with MAIT cell depletion in combined antiretroviral therapy (cART)-naive patients. **a** FLICA caspase-1 induction of total MAIT and CD4^+^ MAIT cells with/without HIV-1 R5 strain JR-CSF challenge for 48 h in PBMCs from HCs (*n* = 9) and TPs (*n* = 9). **b** FLICA caspase-1 induction of MAIT cells with/without HIV-1 R5 strain JR-CSF challenge for 24 h in HCs (*n* = 9) and TPs (*n* = 9) PBMCs activated by αCD3 plus αCD28 antibodies, PFA-fixed *E. coli*, and IL-12 plus IL-18, respectively. **c** FLICA caspase-1 induction of MAIT cells from healthy donors (*n* = 10) incubated for 48 h with HCs’ plasma and TPs’ plasma with CD4^+^ T cell count ≥ 350 cells/µl and TPs’ plasma with CD4^+^ T cell count < 350 cells/µl, respectively. **d** Quantification of plasma IL-12p70 (left panel) and IL-18 (right panel) in HCs (grey, *n* = 33), TPs (total *n* = 69, CD4^+^ T cell count ≥ 350 cells/µl subgroup marked by pink, *n* = 29, CD4^+^ T cell count < 350 cells/µl subgroup marked by red, *n* = 40). Different colors represent different subgroups. Data are expressed as *M* (*Q*_1_, *Q*_3_). ^**^P < 0.01, ^***^P < 0.001, ^****^P < 0.0001, ns non-signifcant. The Wilcoxon signed-rank test was used for matched paired *t*-test (**a** and **b**), Mann–Whitney *U* test (**c** and **d**). The lower detection limit of plasma IL-12p70 by using high sensitivity ELISA kit is 7.8 pg/ml. FLICA fluorescently labeled inhibitor of caspases. HC healthy control, TP treatment-naive patient, HIV human immunodeficiency virus, *E. coli Escherichia coli*, IL-12 interleukin-12, IL-18 interleukin-18

HIV-mediated cell death has been extensively studied [33, 40]; however, whether HIV virions can induce MAIT-cell pyroptosis remains unknown. We first utilized the HIV-1 R5 strain (JR-CSF) to challenge PBMCs from HCs. We found that total HC-MAIT cells expressed higher levels of FLICA caspase-1 after the challenge compared to total HC-MAIT cells incubated with conditioned medium only (*P* < 0.01, Fig. 5a). Further analysis revealed that HC-CD4^+^MAIT cells exhibited higher levels of FLICA caspase-1 expression after challenge when compared with their counterparts in CM only (*P* < 0.01, Fig. 5a). Interestingly, TP-MAIT and TP-CD4^+^MAIT cells did not show significant increases in FLICA caspase-1 expression between HIV-1 R5 strain challenge and CM treatment (Fig. 5a).

Next, we found that both HC-MAIT and TP-MAIT cells evoked higher levels of FLICA caspase-1 expression in response to anti-CD3 plus anti-CD28 Ab or PFA-fixed *E. coli* DH5a strain stimulation (*P* < 0.01, Fig. 5b). Interestingly, HC-MAIT cells showed a further increase in the expression of FLICA caspase-1 in combination with HIV-1 R5 strain stimulation under anti-CD3/anti-CD28 Ab (*P* < 0.01, Fig. 5b) or PFA-fixed *E. coli* DH5a strain stimulation (*P* < 0.01, Fig. 5b), whereas no significant increase in FLICA caspase-1 expression was observed in TP-MAIT cells under the same combined stimulation (Fig. 5b). IL-12 plus IL-18 is a conventional TCR-independent trigger. We also found that both HC-MAIT and TP-MAIT cells expressed higher levels of FLICA caspase-1 in response to IL-12/IL-18 stimulation (*P* < 0.01, Fig. 5b). Similarly, HC-MAIT cells increased FLICA caspase-1 expression after combined IL-12/IL-18 with HIV-1 R5 strain stimulation (*P* < 0.01, Fig. 5b), whereas no significant increase in FLICA caspase-1 expression was observed in TP-MAIT cells after the same combined stimulation (Fig. 5b).

We hypothesized that continuous exposure to high viral loads and pro-inflammatory cytokines could potentially induce MAIT-cell pyroptosis. To test this hypothesis, we incubated PBMCs from healthy donors in a medium containing 20% plasma from HCs or TPs. We found that the frequencies of FLICA caspase-1^+^ MAIT cells significantly increased after incubation with plasma from TPs (*P* < 0.01, Fig. 5c). Furthermore, plasma from TPs with CD4^+^ T cell count < 350 cells/µl induced higher frequencies of FLICA caspase-1^+^ MAIT cells in PBMCs from healthy donors than plasma from patients with CD4^+^ T cell count ≥ 350 cells/µl (*P* < 0.01, Fig. 5c). Next, we assessed the levels of IL-12 and IL-18 in the plasma of HCs and patients with chronic HIV infection. While subunit IL-12 p70 could only be detected in 2 out of 33 HCs, it was more abundant in HIV-1 infected patients where it was found at detectable levels in 4 out of 29 TPs with CD4^+^ T cell count ≥ 350 cells/µl and in 9 out of 40 TPs with CD4^+^ T cell count < 350 cells/µl (Fig. 5d). In addition, we found that plasma IL-18 concentration in TP was higher than that in HCs, especially in TPs with CD4^+^ T cell count < 350 cells/µl (*P* < 0.001 or *P* < 0.0001, Fig. 5d). Thus, in the case of persistent HIV-1 infection, dysregulated plasma levels of IL-12 and IL-18 may contribute to MAIT cell activation, and activation-induced pyroptosis of MAIT cells may lead to the loss of MAIT cells in TPs.

### Increased pyroptotic MAIT cells correlate with poor immunological reconstitution in patients undergoing long-term cART

**Fig. 6:**
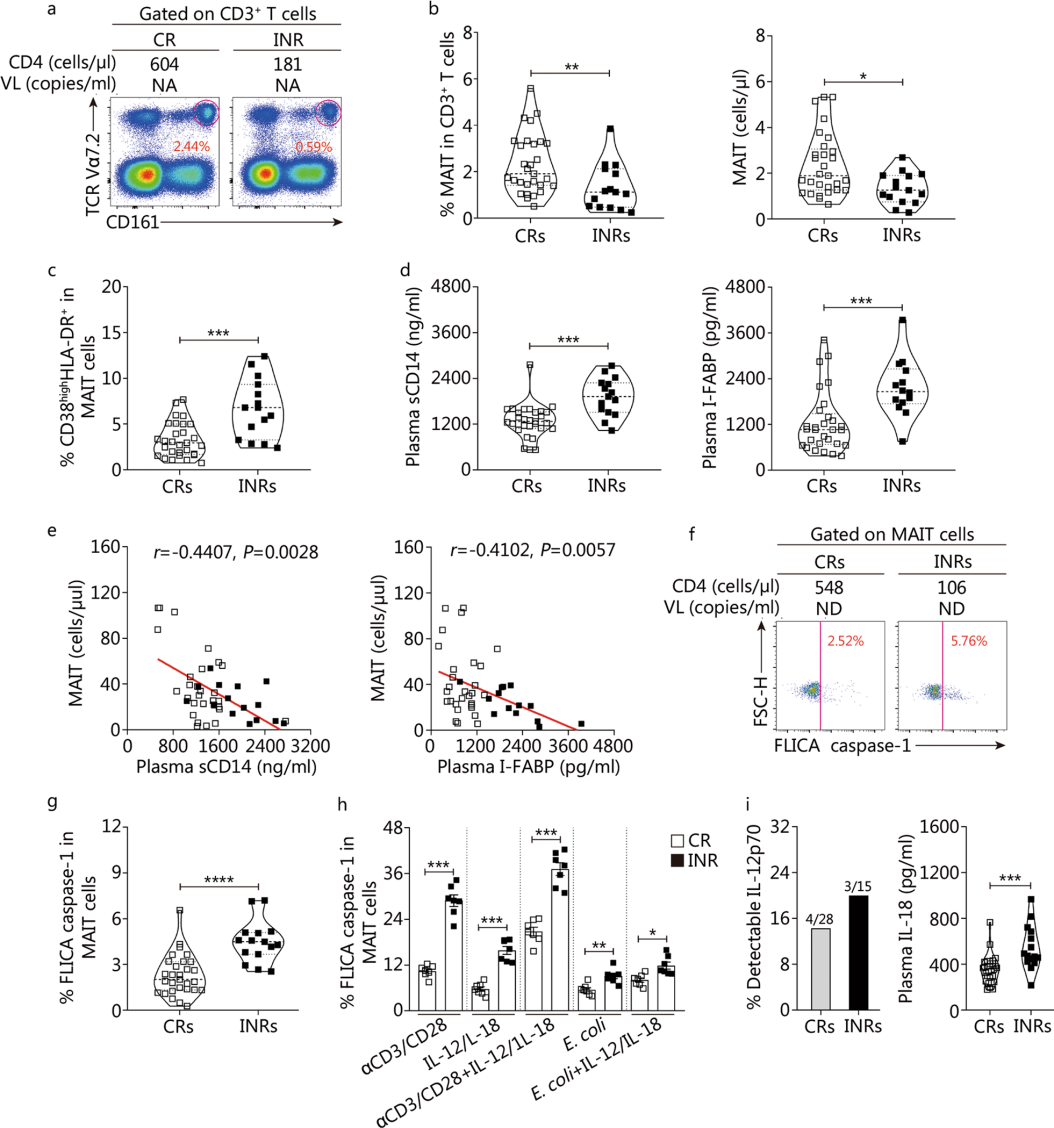
Loss of peripheral mucosal-associated invariant T (MAIT) cells is associated with poor immunological reconstitution in patients with long-term combined antiretroviral therapy (cART). **a** Representative FACS plots from one CR and one INR showing MAIT cells. **b** Pooled data showing MAIT cells percentages and absolute numbers from CRs (hollow squares, *n* = 28) and INRs (solid squares, *n* = 15). **c** Proportion of CD38^high^HLA-DR^+^ MAIT cells from CRs and INRs. **d** Plasma sCD14 levels and I-FABP levels in CRs and INRs. **e** The relationship between MAIT-cell absolute numbers and plasma sCD14 levels or I-FABP levels in CRs and INRs. **f** Representative FACS plots from one CR and one INR showing FLICA-caspase-1^+^ MAIT cells. **g** Pooled data showing frequencies of FLICA-caspase-1^+^ MAIT cells from CRs and INRs. **h** Percentages of FLICA caspase-1^+^ MAIT cells of PBMCs from CRs (*n* = 7) and INRs (*n* = 7) in the presence of indicated stimulations. **i** Quantification of plasma IL-12p70 and IL-18 in CRs and INRs. Each symbol represents a single individual, and data are expressed as *M* (*Q*_1_, *Q*_3_). Mann–Whitney *U* test (**b**, **c**, **d**, **g**, **h** and **i**). Spearman’s correlation test (**e**). *P*-value and Spearman’s Rho value are shown. ^*^P < 0.05, ^**^P < 0.01, ^***^P < 0.001, ^****^P < 0.0001. CRs complete responders, INRs immunological non-responder, VL viral load, NA not available, sCD14 soluble CD14, I-FABP intestinal fatty acid-binding protein, FSC-H forward scatter-height, FLICA fluorescently labeled inhibitor of caspases, IL-12 interleukin-12, IL-18 interleukin-18

Two distinct cohorts of long-term cART-treated patients, namely CRs and INRs, were observed in the clinic [39, 40, 51, 52]. We further investigated the characteristics of MAIT cells in CRs and INRs and found that the frequencies (*P* < 0.01, Fig. 6a, b) and absolute numbers (*P* < 0.05, Fig. 6a, b) of MAIT cells in INRs were significantly lower than those in CRs. Further analysis showed that the frequencies of CD38^high^HLA-DR^+^ INR-MAIT cells were higher than those in CR-MAIT cells (*P* < 0.001, Fig. 6c). We also found that plasma sCD14 (*P* < 0.001, Fig. 6d) and I-FABP levels (*P* < 0.001, Fig. 6d) were significantly elevated in the INRs. Correlation analysis showed that the absolute numbers of MAIT cells were inversely correlated with plasma sCD14 (*r* =  − 0.4407, *P* = 0.0028, Fig. 6e) or I-FABP levels (*r* =  − 0.4102, *P* < 0.0057, Fig. 6e) in patients with long-term cART. Impressively, the frequencies of FLICA caspase-1^+^ INR-MAIT cells were significantly higher than those in CR-MAIT cells (*P* < 0.0001, Fig. 6f, g), indicating higher levels of pyroptosis in INR-MAIT cells. These data suggest that pyroptotic INR-MAIT cells are correlated with the loss of MAIT cells, microbial translocation, and mucosal damage in INR patients.

Next, we detected the responsiveness of CR-MAIT and INR-MAIT cells to TCR-dependent or TCR-independent stimulation. Interestingly, we found that INR-MAIT cells showed a significant increase in FLICA caspase-1 expression when compared with CR-MAIT cells in response to anti-CD3/anti-CD28, IL-12/IL-18, *E. coli*, and combined stimulation in vitro (*P* < 0.05 or *P* < 0.001, Fig. 6h). These data suggest that activation can induce MAIT-cell pyroptosis both in CRs and INRs, and this responsiveness seemed more susceptible in INRs than in CRs. We also assessed the plasma levels of IL-12 and IL-18 and found that IL-12p70 was detected in 4 out of 28 CRs and 3 out of 15 INRs. Additionally, the plasma levels of IL-18 in INRs were significantly higher than those in CRs (*P* < 0.001, Fig. 6i), suggesting that continuous exposure to high levels of proinflammatory cytokines could potentially drive MAIT cell pyroptosis in these patients.

Altogether, these data suggest that increased pyroptotic MAIT cells in long-term cART-treated patients is correlated with poor immunological reconstitution.

## Discussion

Previous studies characterized peripheral blood and gut MAIT cells in HIV-1 infected subjects and demonstrated that: (1) early- and chronic-stage HIV-1/SIV infection leads to quantitative depletion of peripheral or gut MAIT cells [16, 17, 32] and systemic loss of MAIT cells persists despite successful cART [17]; and (2) functional impairment of MAIT cells is associated with reduced defense against microbial infections and endothelial repair capacity [24]. These findings clearly describe the indispensable role of MAIT cells in the context of HIV-1 infection. However, the mechanisms underlying MAIT cell loss and correlative clinical consequences remain poorly understood. Here, we report that chronic HIV-1 infection triggered pyroptosis of MAIT cells in the peripheral blood and gut mucosa of HIV-1 infected patients and increased pyroptotic MAIT cells are associated with disease progression and immune reconstitution. These findings might be important for understanding the pathogenesis of HIV-1 and might be helpful for the development of strategies aimed at recovering immune function in HIV-1 infected patients.

Both innate-like T cells and innate lymphoid cells (ILCs) are severely depleted during acute and chronic HIV-1 infection [53]. However, the mechanisms driving the loss of these cells differ. Previous studies have shown that circulating CD1d-restricted iNKT cells and γδ T cells were abundantly reduced, independent of CD4^+^ T cell count and plasma viral load [54, 55]. van der Vliet et al. [56] showed that the loss of iNKT cells in peripheral blood was due to Fas–Fas ligand-mediated apoptosis and tissue sequestration compared to direct death from HIV-1 infection. In contrast, the death of Vγ2Vδ2 T cells was highly dependent on the p38-caspase-2, -8, and -9 signaling pathways during progression to acquired immune deficiency syndrome (AIDS) [57]. Peripheral ILCs (especially ILC3) in patients with hyperacute HIV infection showed upregulated expression of apoptosis-associated genes. In the chronic stage, circulating ILCs in HIV-1 infected individuals upregulated the expression of CD38 and Fas but not apoptosis-associated genes [58]. In the present study, we comprehensively detected the characteristics of MAIT cells at different disease progression stages and confirmed previous reports that the frequencies and absolute numbers of MAIT cells were decreased in HIV-1 infected patients. Importantly, we found that frequencies and absolute numbers of MAIT cells were decreased in a cohort of ECs. The frequency and absolute number of MAIT cells were further decreased in one EC patient from this cohort after HIV-1 massive replication. These properties of MAIT cells may represent a previously uncharacterized mechanism of MAIT cell depletion during HIV-1 infection. Interestingly, we found that MAIT cells showed a higher pyroptotic phenotype in HIV-1 infected patients, indicating that the preferential pyroptosis of MAIT cells is associated with MAIT cell loss. There is evidence that supports this notion: first, we found that the frequency of pyroptotic MAIT cells was negatively correlated with the absolute numbers of MAIT cells, which often positively correlated with CD4 cell count in HIV-1 infected patients. Although no correlation was observed between the frequencies of MAIT or pyroptotic MAIT cells with viral load, we found that the frequencies of CD4^+^ MAIT and pyroptotic CD4^+^ MAIT cells showed a significant inverse correlation with viral load. Furthermore, increased frequencies of pyroptotic CD4^+^ MAIT cells from one EC lost control were observed with a decrease in frequency and quantity. Second, MAIT cells in the gut mucosa of HIV-1 infected patients exhibited a stronger active GSDMD signal than in the gut mucosa of HIV-1 negative individuals. Third, the frequencies of pyroptotic MAIT cells in INRs were higher than those in CRs. Thus, the observed pyroptotic MAIT cells may lead to a decrease in MAIT cells in HIV-1 patients and represent an HIV-1 specific and nonspecific phenomenon.

The mechanisms leading to the pyroptosis of MAIT cells during HIV-1 infection need to be elucidated. Our scRNA-seq and flow cytometry results indicated that circulating MAIT cells or pyroptotic MAIT cells from TP patients were hyperactivated. We also observed that both the frequencies of MAIT and pyroptotic MAIT cells were significantly positively correlated with markers of systemic T cell activation in TPs. In addition, the activation state of MAIT cells in INRs was higher than that in CRs. MAIT cell activation is tightly regulated by TCR-dependent and by a variety of cytokines, such as IL-12 and IL-18. We found that both HC-MAIT cells and TP-MAIT cells increased active caspase-1 expression under TCR-dependent or TCR-independent stimulation in vitro. Interestingly, HC-MAIT cells showed a greater increase in active caspase-1 expression under TCR-dependent or TCR-independent stimulation in the presence of HIV-1 virion, whereas TP-MAIT cells showed poorer responsiveness to the superimposed stimulation, which may be partially due to the profound decrease in CD4^+^ MAIT in total MAIT cells of TPs because active caspase-1 expression in CD4^+^ MAIT cells can be induced by HIV virion challenge. Importantly, INR-MAIT cells showed elevated active caspase-1 expression in response to TCR-dependent or TCR-independent stimulation or superimposed stimulation in vitro. We then investigated the cause of MAIT cell activation in HIV-1 infected patients. In agreement with previous studies, we detected higher levels of IL-12 and IL-18 in the plasma of HIV-1 infected patients. Such elevations may be responsible for elevated MAIT cell pyroptosis because of their ability to induce MAIT activation.

There are some potential limitations to this study: first, the colorectal tissues were obtained from HIV-1 infected patients with rectal or colon cancer, which may lead to alterations of MAIT cells even in the absence of HIV; therefore, it is necessary to enroll HIV-1 infected patients without cancer to further investigate the characteristics of tissue-resident MAIT cells. Second, the combinations of cell-surface markers, such as CD3, TCR Vα7.2, and CD161, as well as surrogate markers such as MDR1 or IL-18α plus TCR Vα7.2, were used to identify MAITs in peripheral blood or tissues before MR1 tetramer availability. Cells expressing these markers were enriched for MAIT cells but do not represent 100% pure MAIT cells; therefore, it would be ideal to use the MR1 tetramer to identify MAIT cells in the context of HIV infection. Finally, further studies are needed to determine the proper approach to reducing MAIT cell pyroptosis in the future.

## Conclusions

**Fig. 7:**
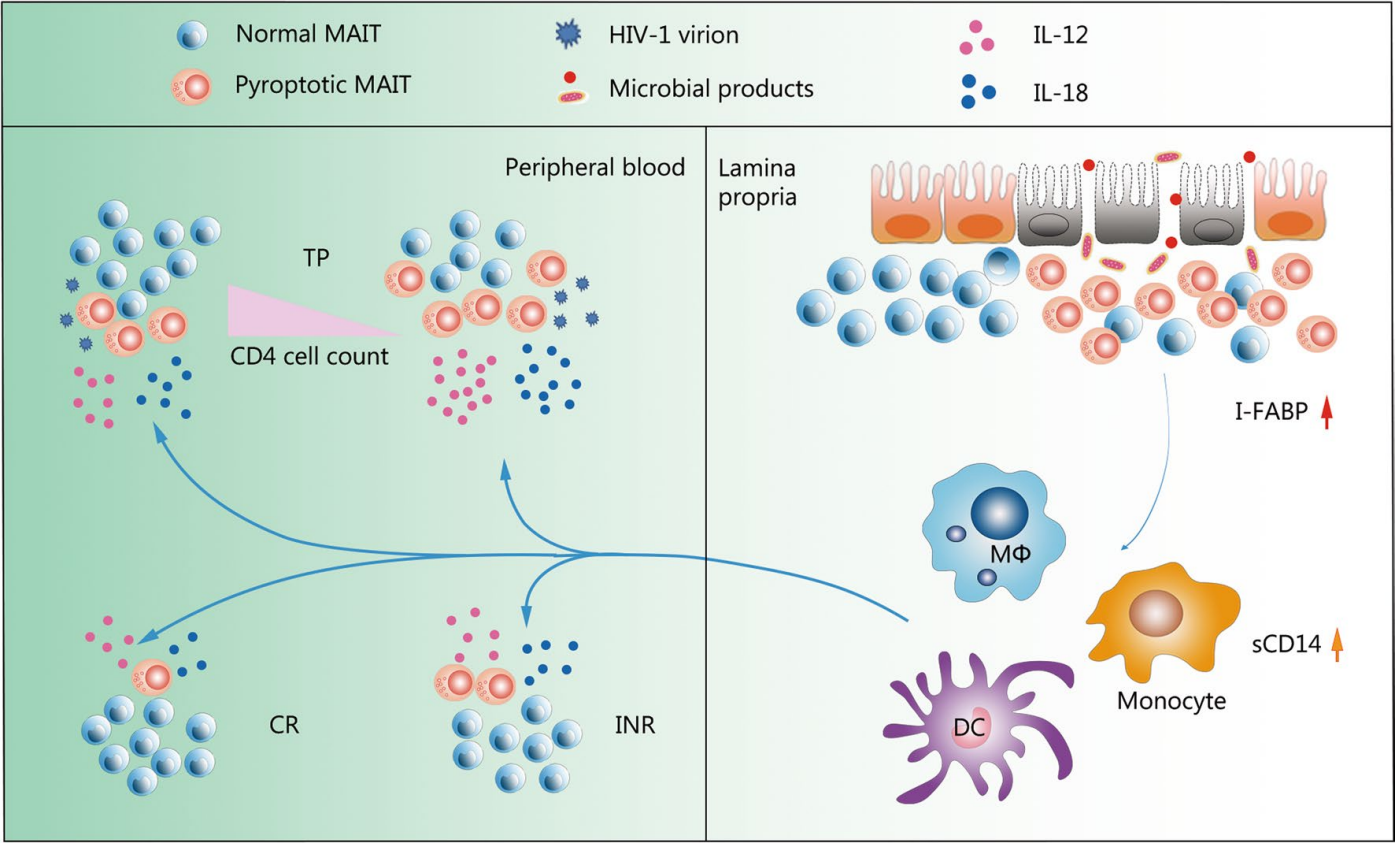
Schematic representation of activation-induced pyroptosis of mucosal-associated invariant T (MAIT) cells in chronic human immunodeficiency virus type 1 (HIV-1) infection. Following chronic HIV-1 infection, MAIT cells decreased in peripheral blood and homed to the gut mucosa due to activation-induced pyroptosis in response to multiple stimuli, including HIV-1 virions, IL-12, and IL-18 stimulation. This correlates with HIV-1 disease progression and severity of microbial translocation. However, MAIT-cell pyroptosis decreased in CRs but remained higher in INRs following successful combined antiretroviral therapy. TP treatment-naive patient, CR complete responder, INR immunological non-responder, IL-12 interleukin-12, IL-18 interleukin-18, sCD14 soluble CD14, I-FABP intestinal fatty acid-binding protein, DC dendritic cells

Taken together, it is reasonable to speculate that MAIT cells during chronic HIV-1 infection might be persistently activated and driven in pyroptosis in response to multiple strikes, such as HIV-1 virion, IL-12, and IL-18 stimulation, demonstrated by MAIT cells being decreased in peripheral blood but enriched in the gut mucosa with an outstanding pyroptotic phenotype. As a result, the damaged gastrointestinal tract is difficult to repair, meaning that more microbes and/or microbial products can enter the blood circulation and cause systemic immune activation, increased innate cytokines release, and increased MAIT cell loss (Fig. 7). This bad circulation loop may also work in patients with INR and influence immune recovery during cART. These findings have important implications for evaluating MAIT cell pyroptosis as novel therapeutic targets to improve HIV-1 associated immunopathology.

## Supplementary Information

**Additional file 1:** **Fig. S1.** Gating strategy of mucosal-associated invariant T (MAIT) cells. **Fig. S2.** Correlation analysis of mucosal-associated invariant T (MAIT) cell frequencies with CD4^+^ T-cell counts (**a**), plasma human immunodeficiency virus (HIV) viral load (**b**) and the frequencies of CD38^high^HLA-DR^+^-expressing MAIT cells (**c**). **Fig. S3.** Workflow and quality-control of scRNA-seq. **Fig. S4.** Increased mucosal-associated invariant T (MAIT) cells in colorectal mucosa tissues from human immunodeficiency virus type 1 (HIV-1) infected patients.

**Additional file 2:** **Table S1.** Antibodies for flow cytometry. **Table S2.** Antibodies for immunohistochemistry. **Table S3.** Characteristics of enrolled HIV-1 infected patients in single-cell RNA sequencing.

## Data Availability

Not applicable.

## Abbreviations

AIDS: Acquired immune deficiency syndrome
cART: Combined antiretroviral therapy
CCR9: C–C motif chemokine receptor 9
CRs: Complete responders
CXCR3: CXC-chemokine receptor 3
DEG: Differential expression genes
ECs: Elite controllers
*E. coli*: *Escherichia coli*
FLICA: Fluorescently labeled inhibitor of caspases
GSDMD: Gasdermin-D
GSDME: Gasdermin-E
GSEA: Gene set enrichment analysis
HCs: Healthy controls
HIV-1: Human immunodeficiency virus type 1
HLA: Human leukocyte antigen
IFN-γ: Interferon-γ
I-FABP: Intestinal fatty acid-binding protein
IL-12: Interleukin-12
ILCs: Innate lymphoid cells
iNKT: Innate natural killer T
INRs: Immunological non-responders
MAIT cells: Mucosal-associated invariant T cells
PBMCs: Peripheral blood mononuclear cells
QC: Quality control
SIV: Simian immunodeficiency virus
scRNA-seq: Single-cell RNA sequencing
sCD14: Soluble CD14
TCR: T-cell receptor
TPs: Treatment-naive patients
TNF-α: Tumor necrosis factor-α
