# Supplementary material for: Activation-induced pyroptosis contributes to the loss of MAIT cells in chronic HIV-1 infected patients
Source: Mil Med Res. 2022 May 27;9:24. doi: 10.1186/s40779-022-00384-1 (PMC9137088; doi:10.1186/s40779-022-00384-1)
Supplement: Supplementary file 1 — Additional file 1: Fig. S1. Gating strategy of mucosal-associated invariant T (MAIT) cells. Fig. S2. Correlation analysis of mucosal-associated invariant T (MAIT) cell frequencies with CD4+ T-cell counts (a), plasma human immunodeficiency virus (HIV) viral load (b) and the frequencies of CD38highHLA-DR+-expressing MAIT cells (c). Fig. S3. Workflow and quality-control of scRNA-seq. Fig. S4. Increased mucosal-associated invariant T (MAIT) cells in colorectal mucosa tissues from human immunodeficiency virus type 1 (HIV-1) infected patients. [file 40779_2022_384_MOESM1_ESM.pdf]

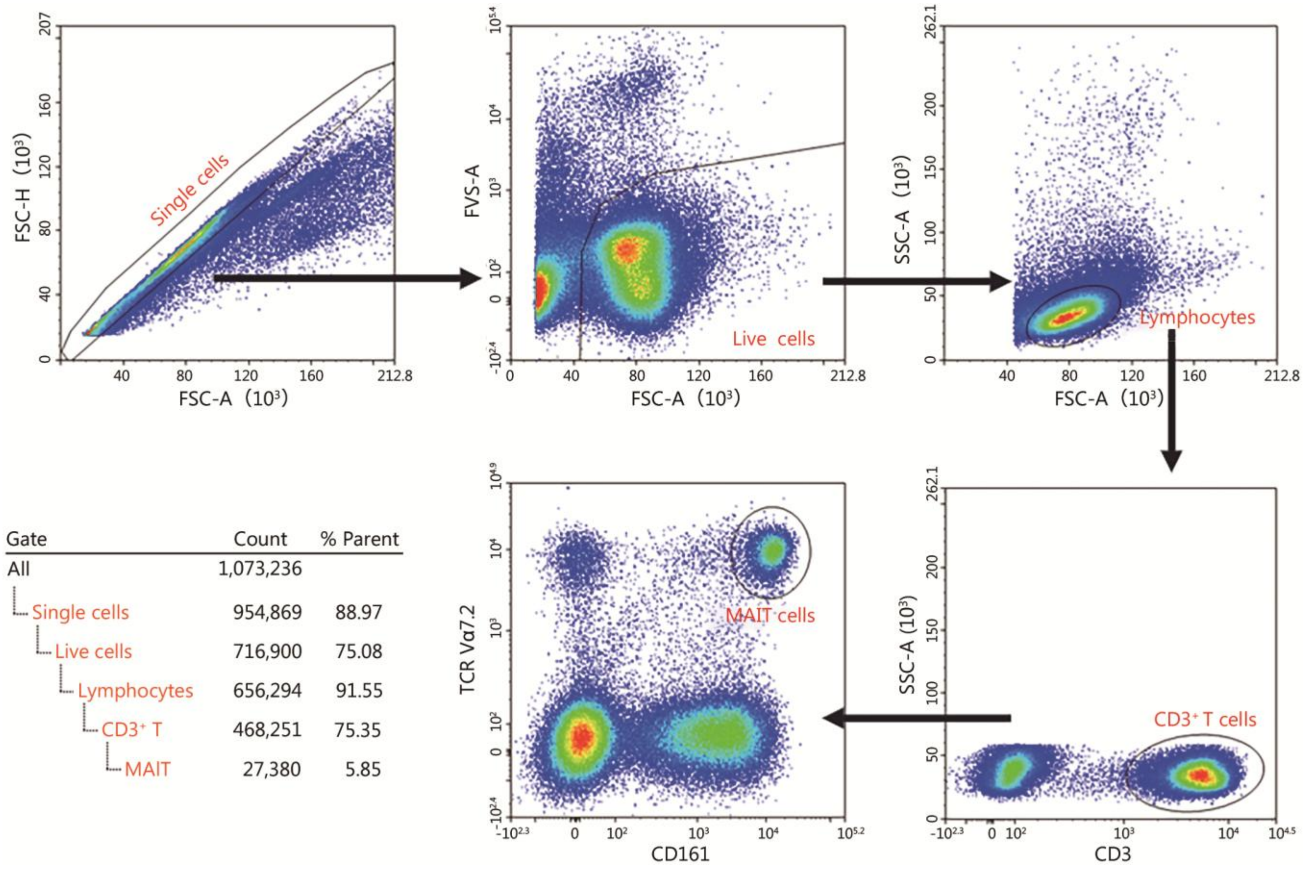

Fig. S1 Gating strategy of mucosal-associated invariant T (MAIT) cells. MAIT cells were gated on $CD3^{+}$ $CD161^{high}$ TCR $V\alpha7.2^{+}$ cells. FSC-H forward scatter-height, FVS-A fixable viability stain-area, FSC-A forward scatter-area, TCR T-cell receptor, SSC-A side scatter-area

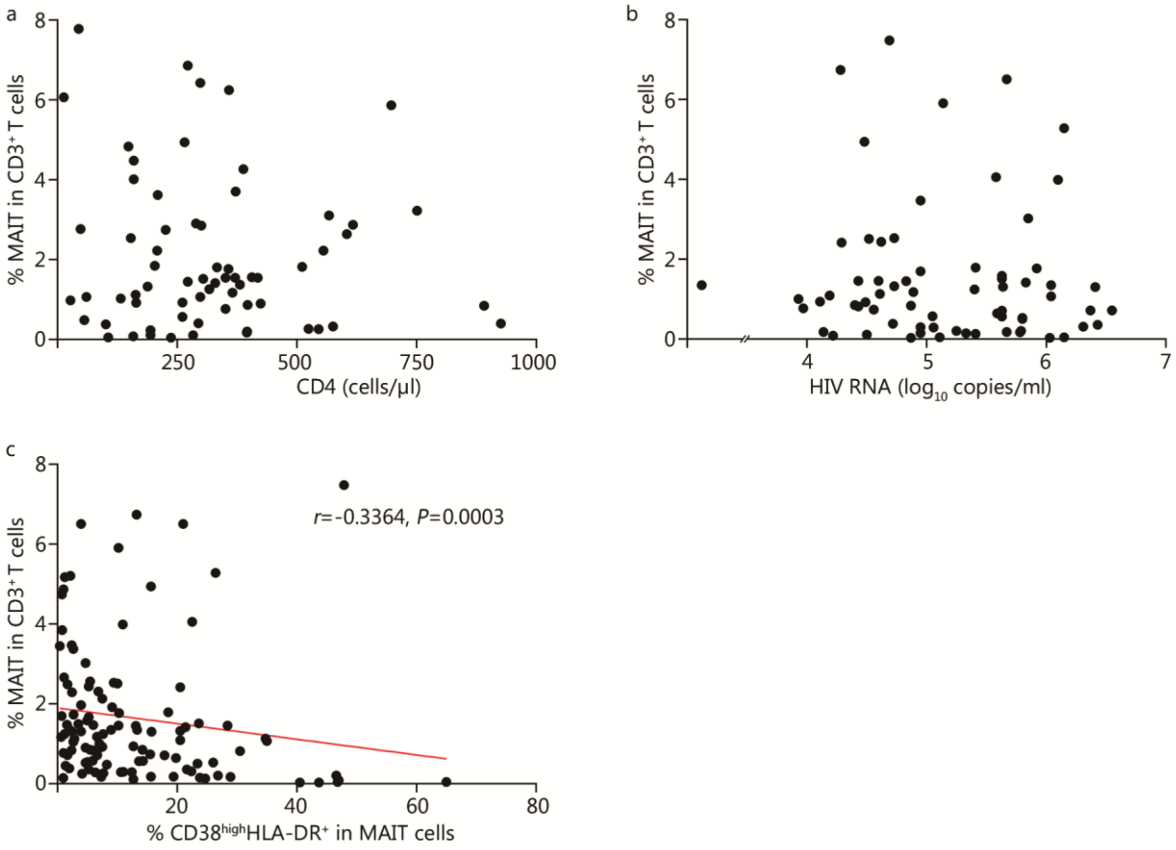

Fig. S2 Correlation analysis of mucosal-associated invariant T (MAIT) cell frequencies with $CD4^{+}$ T-cell counts **(a)**, plasma human immunodeficiency virus (HIV) viral load **(b)** and the frequencies of $CD38^{high}HLA\text{-}DR^{+}$-expressing MAIT cells **(c)**. Each symbol represents a single individual. Associations were evaluated by using Spearman rank correlation test. *P*-value and Spearman's Rho value are shown

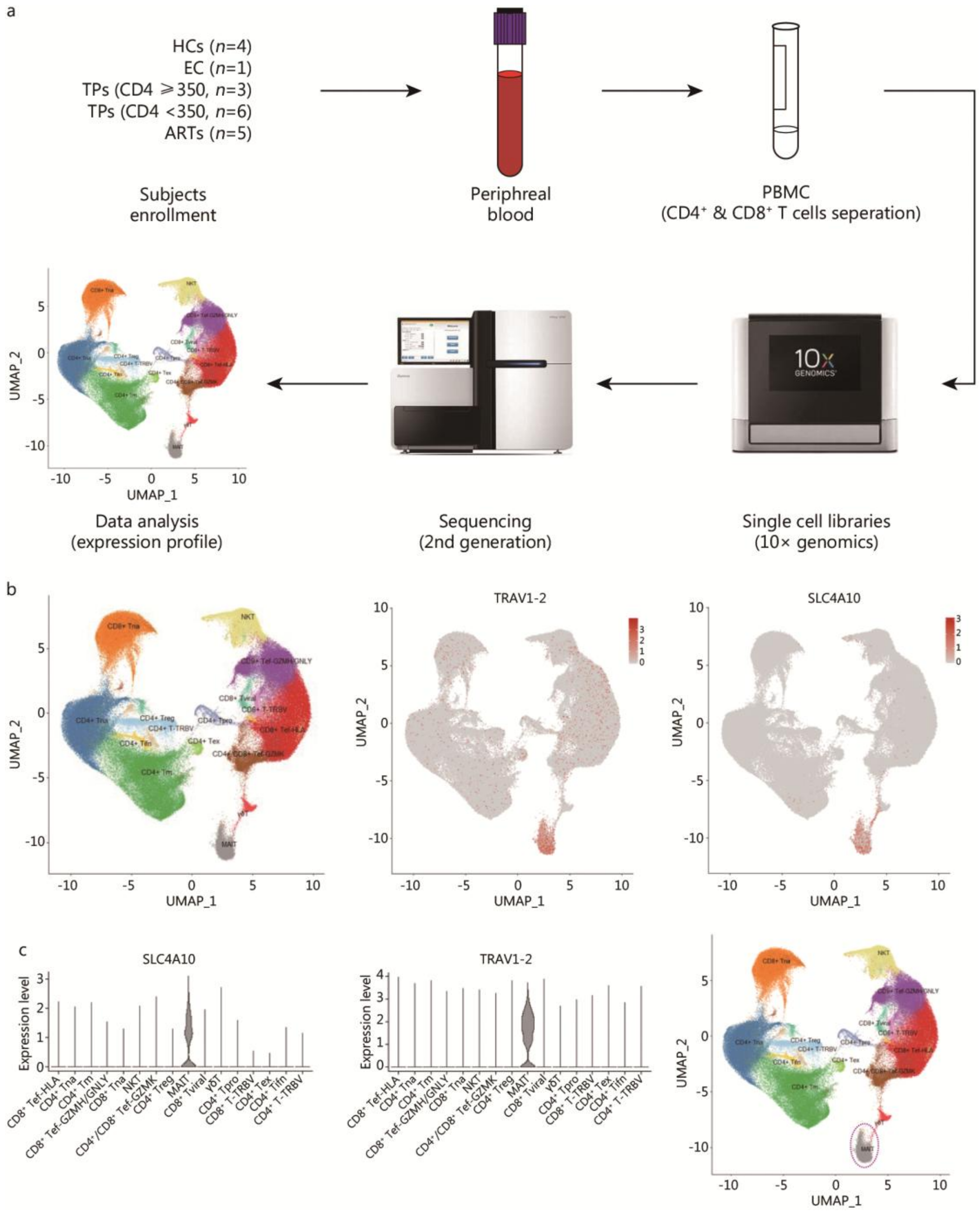

Fig. S3 Workflow and quality-control of scRNA-seq. **a** Workflow of the scRNA-seq experiment, including subjects grouping, sample collection, CD4^{+} T cells and CD8^{+} T cells purification, single cell cDNA libraries construction, second generation sequencing (performed by CapitalBioTechnology, Beijing, China) and bioinformatic analysis. **b** Clustering of 182,670 cells from all 19 samples in this study. Each dot corresponds to a single cell, colored according to cell types. **c** Violin plot of two canonical markers' expression levels (SLC4A10 and TRAV1-2) across cell clusters. Red circle in the UMAP highlights MAIT cell cluster. HCs healthy controls, EC elite controller, TPs treatment-naïve patients, ART antiretroviral therapy, PBMC peripheral blood mononuclear cell

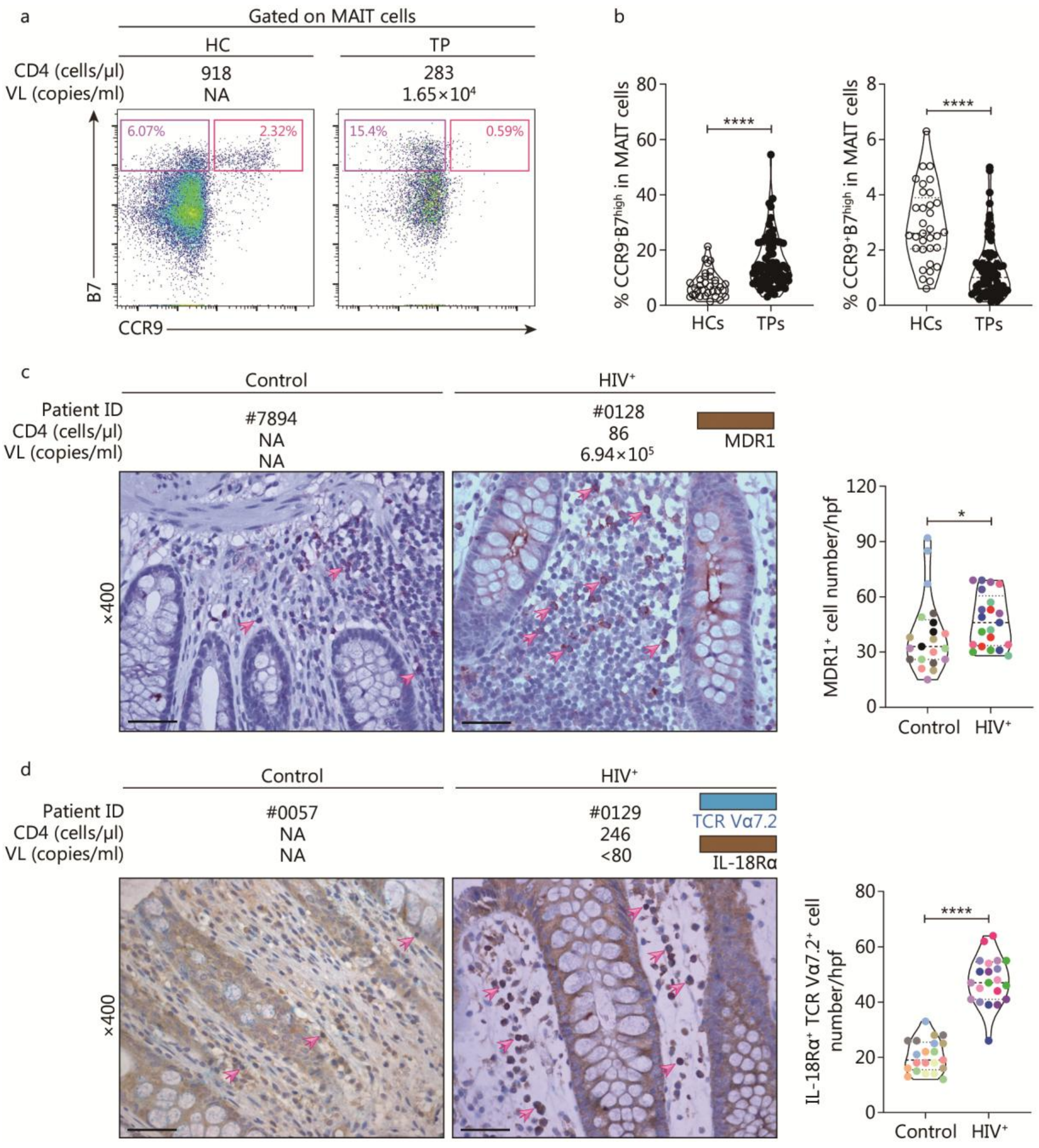

Fig. S4 Increased mucosal-associated invariant T (MAIT) cells in colorectal mucosa tissues from human immunodeficiency virus type 1 (HIV-1) infected patients. **a** Representative FACS plots from one HC and one TP displaying CCR9 and integrin β7 expression. **b** Pooled data showing the frequencies of $CCR9^{-}$integrin $β7^{high}$ and $CCR9^{+}$integrin $β7^{high}$ MAIT cells from HCs (hollow circles, *n* = 33) and TPs (solid circles, total *n* = 69). **c** Representative immunohistochemistry images (×400) and quantification of MAIT cells in colorectal mucosa tissues from HIV-negative controls (*n* = 7) and HIV^{+} patients (*n* = 7) single-stained with anti-MDR1. Arrowheads highlight canonical MDR1 only positive cells. **d** Representative immunohistochemistry images (×400) and quantification of MAIT cells in colorectal mucosa tissues from HIV-negative (*n* = 7) and HIV^{+} patients (*n* = 7)

co-stained with anti-IL-18Rα and anti-TCR Vα7.2. Arrowheads highlight canonical IL-18Rα$^+$TCR Vα7.2$^+$ double-positive cells. Each color in the pooled data (**c** and **d**) of immune single or double stains represents one single subject, and each dot represents single or double-positive cell numbers counted in a single high-power field (hpf, ×400). Each symbol represents a single individual. Data are expressed as M (Q_1, Q_3). $^*P < 0.05$, $^{****}P < 0.0001$. Scale bar of hpf = 50 μm. Mann-Whitney U test (**b-d**). HC healthy control, TP treatment-naïve patient, VL viral load, NA not available, CCR9 C-C motif chemokine receptor 9, HIV human immunodeficiency virus, IL-18 interleukin-18, TCR T-cell receptor
