# Supplementary material for: Activation-induced pyroptosis contributes to the loss of MAIT cells in chronic HIV-1 infected patients
Source: Mil Med Res. 2022 May 27;9:24. doi: 10.1186/s40779-022-00384-1 (PMC9137088; doi:10.1186/s40779-022-00384-1)
Supplement: Supplementary file 2 — Additional file 2: Table S1. Antibodies for flow cytometry. Table S2. Antibodies for immunohistochemistry. Table S3. Characteristics of enrolled HIV-1 infected patients in single-cell RNA sequencing. [file 40779_2022_384_MOESM2_ESM.pdf]

Table S1 Antibodies for flow cytometry

Markers	Clones	Fluorochromes	Cat. No.	Companies
CD3	OKT3	Brilliant Violet 510™	317332	Biolegend, USA
CD3	SK7	APC/Cy7	557832	Biolegend, USA
CD4	SK3	PerCP	247234	BD Biosciences, USA
CD161	HP-3G10	PE	339904	Biolegend, USA
TCR Vα7.2	3C10	Brilliant Violet 421™	351716	Biolegend, USA
CD38	HIT2	APC	555462	BD Biosciences, USA
HLA-DR	L243	PerCP	347364	BD Biosciences, USA
PD-1	EH12.2H7	PE	329906	Biolegend, USA
IFN-γ	4S.B3	PE/Cy7	557844	BD Biosciences, USA
TNF-α	MAb11	FITC	502912	Biolegend, USA
CCR9	L053EB	PE/Cy7	358910	Biolegend, USA
Integrin B7	FIB504	APC	555943	BD Biosciences, USA

TCR T-cell receptor, *HLA* human leukocyte antigen, *PD-1* programmed death-1, *IFN-γ* interferon-γ, *TNF*-α tumor necrosis factor-α, *CCR9* C-C motif chemokine receptor 9

Table S2 Antibodies for immunohistochemistry

Names	Clones	Cat. No.	Dilution	Companies
Rabbit anti-human MDR1	EPR10364-57	ab170904	1:100	Abcam, UK
Goat anti-human IL-18Rα	Polyclonal	AF840	1:250	R&D systems, USA
Mouse anti-human TCR Vα7.2	3C10	351702	1:500	Biolegend, USA
Mouse anti-human CD4	UMAB64	ZM-0418	1:500	Origene, USA

MDR1 multi-drug resistance protein 1, *IL-18* interleukin-18, *TCR* T-cell receptor

Table S3 Characteristics of enrolled HIV-1 infected patients in single-cell RNA sequencing

HIV status/Patient ID	Gender	Age (years)	$CD4^+$ T cell count (cells/μl)	$CD8^+$ T cell count (cells/μl)	Plasma viral load (copies/ml)	cART regimen
HCs (*n* = 4)						
HC01	Male	27	1134	940	NA	NA
HC02	Male	31	525	352	NA	NA
HC03	Male	27	816	634	NA	NA
HC04	Male	28	822	621	NA	NA
EC (*n* = 1)						
EC01	Male	26	627	822	NA	NA
TPs (*n* = 9)						
$CD4^{high}$ (CD4 ≥ 350, *n* = 3)						
TP-H01	Male	30	448	935	13,200	NA
TP-H02	Male	40	383	1659	280,000	NA
TP-H03	Male	23	541	874	47,200	NA
$CD4^{low}$ (CD4 < 350, *n* = 6)						
TP-L01	Male	21	302	1450	32,200	NA
TP-L02	Male	25	266	1049	13,500	NA
TP-L03	Male	26	348	1352	32,700	NA

TP-L04	Male	23	176	661	126,000	NA
TP-L05	Male	43	289	854	190,000	NA
TP-L06	Male	30	198	998	1230,000	NA
ARTs (cART-treated patients, *n* = 5)						
ART01	Male	24	457	604	< LOD	3TC/EFV/TDF
ART02	Male	29	459	618	< LOD	3TC/AZT/NVP
ART03	Male	32	311	460	< LOD	3TC/EFV/TDF
ART04	Male	30	627	746	< LOD	3TC/EFV/TDF
ART05	Male	25	459	1047	< LOD	3TC/EFV/TDF

HIV human immunodeficiency virus, *cART* combined antiretroviral therapy, *HCs* healthy controls, *NA* not applicable, *EC* elite controller, *TPs* treatment-naive patients, *ART* antiretroviral therapy, *<LOD* below the limit of detection of 80 copies/ml, *3TC* lamivudine, *EFV* efavirenz, *TDF* tenofovirdisoproxil, *AZT* azidothymidine, *NVP* nevirapine
